# Smart sensing-enabled risk-aware nitrogen prescriptions via conformal profit bounds for precision agriculture

**DOI:** 10.3389/fpls.2026.1821003

**Published:** 2026-04-27

**Authors:** Abuzar Khan, Ahmad Junaid, Abid Iqbal, Sajid Iqbal, Latifah Almuqren, Ghassan Husnain, Syed Hashim Raza Bukhari, Mohammed Al-Naeem

**Affiliations:** 1 Department of Computer Science, CECOS University of IT and Emerging Sciences, Peshawar, Pakistan; 2 Department of Computer Engineering, College of Computer Sciences and Information Technology, King Faisal University, Al-Ahsa, Saudi Arabia; 3 Department of Information Systems, College of Computer Sciences and Information Technology, King Faisal University, Al-Ahsa, Saudi Arabia; 4 Department of Information Systems, College of Computer and Information Sciences, Princess Nourah bint Abdulrahman University, Riyadh, Saudi Arabia; 5 Department of Computer Networks Communications, College of Computer Sciences and Information Technology, King Faisal University, Al-Ahsa, Saudi Arabia

**Keywords:** nitrogen prescriptions, plant science, precision agriculture, smart sensing, sustainable plant-environment management

## Abstract

**Introduction:**

Smart sensing is becoming central to plant science because it supports crop management decisions that reflect dynamic plant–environment interactions rather than field-level averages. Nitrogen fertilization is one of the most important decisions in precision agriculture, but site-specific prescriptions are often difficult to trust because spatial and seasonal variability can make point recommendations unstable.

**Methods:**

We propose an uncertainty-aware nitrogen prescription framework that combines yield-response modeling with conformal prediction intervals and propagates uncertainty into profit bounds under an environmental proxy penalty. Nitrogen rates are selected by maximizing the lower confidence bound of profit, and the framework allows abstention when competing rates are indistinguishable or uncertainty is excessive. Evaluation used a leakage-free group split of 10,000 samples from a Kaggle yield dataset.

**Results:**

The selected tree-ensemble model achieved RMSE 1.531 and MAE 1.214. Conformal intervals reached empirical coverage of 0.912 at the 0.90 target and 0.963 at the 0.95 target. Expected-profit optimization gave mean profit 4.68 with mean N 121.34, whereas the risk-aware strategy gave mean profit 4.54 with mean N 112.07 and lower variability. Abstention withheld recommendations for 18.4% and 27.9% of cases at 0.90 and 0.95 coverage.

**Discussion:**

The framework supports conservative, trustworthy nitrogen decisions and promotes practical nutrient stewardship in variable agricultural fields.

## 1 Introduction

Plant science is increasingly central to addressing food security, environmental degradation and the need for more sustainable agricultural production under climate and resource constraints (Mgendi, 2024). In this context, smart sensing has emerged as a key enabler of modern crop management by allowing plant status, environmental variability and management responses to be observed and acted upon at fine spatial and temporal scales (Al-Gaadi et al., 2023). Precision agriculture (PA) represents one of the most visible engineering realizations of this transition, integrating sensing, geospatial technologies, predictive modeling and variable-rate actuation to optimize inputs while improving productivity, profitability and sustainability (Mgendi, 2024; Zeb et al., 2025).

Within plant science, sustainable phytoprotection depends not only on direct pest and disease interventions but also on sound management of plant-environment interactions that influence crop vigor, stress exposure and environmental burden (Yin et al., 2025). Nitrogen management is especially important in this regard because nitrogen strongly affects plant growth and canopy condition, while excess or poorly targeted application contributes to leaching, runoff and other agroecosystem risks (Shi-ting et al., 2023; Łukowiak et al., 2024). For this reason, smart sensing-enabled nitrogen management can be understood as a phytoprotection-relevant decision problem: it uses plant and environmental information to support healthier crop development while reducing environmentally harmful over-application.

Despite major progress in sensing and automation, a critical gap remains between predictive capability and decision trustworthiness (Khan et al., 2026a). Contemporary PA systems can collect large volumes of information from soil, terrain, weather and crop-status proxies, yet decision-support systems often continue to deliver point recommendations without expressing whether those recommendations are sufficiently reliable under real-world variability (Brugler et al., 2024; Alam et al., 2025). This limitation is especially problematic at the within-field scale, where local errors may propagate across large managed areas and where atypical weather, management shifts or noisy measurements can undermine user confidence in variable-rate prescriptions (Yeo and Keske, 2024).

### 1.1 Smart sensing, plant-environment interactions and nitrogen management under

### within-field variability

Nitrogen is a high-impact management lever because its effects are mediated by spatially variable plantsoil-atmosphere interactions (Yin et al., 2025). Within a single field, soil properties, terrain position, water availability and crop status may vary substantially, causing strong differences in yield response and in the likelihood of environmental nitrogen loss (Adhikari et al., 2023; Song et al., 2026). Nitrogen recovery rates are often approximately 50%, which means a considerable fraction remains in soil pools or is lost through volatilization, denitrification, leaching or runoff (Shi-ting et al., 2023). These losses are not spatially uniform, making single field-level recommendations inadequate for sustainable management (de Lara et al., 2023).

a. Sensor-based variable-rate real-time fertilizer applicator, tractor-mounted (Mirzakhaninafchi et al., 2022). b. UAV-based precision spraying example (Hanif et al., 2022).

Smart sensing offers the observational basis needed to address this challenge. Remote sensing, proximal sensing, yield monitoring, weather observations and geospatial soil information allow plant and environmental heterogeneity to be characterized in ways that support site-specific intervention (Ravikumar et al., 2024). In this sense, precision nitrogen management is not only an optimization problem but also a plant science sensing problem: the objective is to translate sensed crop and environmental signals into interventions that improve nutrient-use efficiency and reduce ecological burden. However, although sensing and actuation capabilities have advanced rapidly, decision quality under uncertainty remains a major limitation (Faisal et al., 2025). Existing methods still predominantly emphasize point yield prediction or a point economic optimal nitrogen rate (EONR) (Leitão et al., 2025). Even when such methods perform well on average, they may fail under previously unobserved seasons, new regions or atypical weather conditions, thereby weakening trust in smart sensing-driven recommendations (Joshi et al., 2024; Alzahrani et al., 2025).

### 1.2 From sensing-informed point prescriptions to risk-aware, selective recommendations

This study addresses the gap between sensing-informed prediction and trustworthy decision support by introducing an uncertainty-aware prescription framework for nitrogen management that is explicitly aligned with smart sensing needs in plant science. Yield is modeled as a function of contextual plant and environmental features together with nitrogen rate and conformal prediction (CP) is used to produce calibrated yield intervals with target coverage under leakage-free group evaluation (Angelopoulos and Bates, 2023). These yield intervals are propagated into profit bounds through a transparent economic model that also includes an environmental proxy penalty. Nitrogen rates are then selected by maximizing the lower confidence bound (LCB) of profit, providing a conservative alternative to point EONR prescriptions (Wu et al., 2025). In addition, selective recommendation is implemented through abstention rules that withhold prescriptions when uncertainty is excessive or when competing rates are statistically indistinguishable. This makes the system operationally useful in smart sensing workflows, where uncertain cases may be redirected to additional sensing, further sampling, conservative defaults or targeted strip trials (Khan et al., 2026c).

### 1.3 Objectives and contributions

The objective of this study is to develop a smart sensing-compatible decision framework for site-specific nitrogen management that remains reliable under within-field variability by explicitly incorporating uncertainty into plant-environment decision making. More specifically, yield uncertainty is calibrated using CP, yield intervals are converted into profit intervals through an explicit profit model with an environmental proxy penalty and nitrogen rates are selected through LCB profit optimization so that recommendations are conservative, stable and risk-aware rather than purely expectation-driven. Reliability is further operationalized through selective abstention, enabling the system to indicate when available sensing and contextual information are insufficient to support a confident recommendation.

This work contributes to the smart sensing and plant science literature in three ways. First, it introduces a decision-focused pipeline that links smart sensing-relevant crop response estimation to profit-based nitrogen prescriptions at the within-field scale, moving beyond point prediction toward risk-aware management. Second, it applies CP as a model-agnostic calibration layer that provides deployment-relevant coverage guarantees and can be integrated with different yield-response models used in sensing-driven agricultural decision support. Third, it evaluates the framework using decision-centered criteria that are meaningful for sustainable management, including profitability, prescription stability and an explicit reliability-versus-coverage trade-off enabled by abstention. Collectively, these contributions position nitrogen recommendation not merely as an optimization task, but as a trustworthy smart sensing problem within plant-environment management and sustainable phytoprotection.

**Supplementary Figure 2** summarizes the end-to-end workflow of this study, linking data preparation and model selection to uncertainty calibration and risk-aware decision making. The remainder of this paper is organized as follows. Section 2 reviews related work on smart sensing for nitrogen management, uncertainty quantification and reliable recommendation under plant-environment variability. Section 3 presents the proposed framework, including CP, profit interval construction and LCB decision rules. Section 4 describes the dataset, group split protocol, baselines and evaluation settings. Section 5 reports predictive and decision outcomes and Section 6 interprets the findings through robustness and sensitivity analyses. Finally, Section 7 concludes the paper and outlines future research directions.

## 2 Literature review

Research on precision nitrogen management sits at the intersection of plant science, smart sensing and decision engineering. The central premise is that plant response and nitrogen-loss risk vary across space and time and that these variations can be observed through sensing and translated into more targeted interventions (Pawase et al., 2023; Khan et al., 2026a). The framing of sustainable phytoprotection because better characterization of plantenvironment interactions can improve crop health management while reducing environmentally harmful over-application. Nevertheless, an important methodological gap persists (Adereti et al., 2024). Many studies develop accurate predictive models or produce spatially explicit nitrogen recommendations, yet far fewer address whether those recommendations remain trustworthy under uncertainty and fewer still provide a principled mechanism for withholding action when confidence is low (Barons et al., 2024; Husnain et al., 2026). This review therefore focuses on the transition from sensing-informed point prescriptions to uncertainty-aware, reliability-centered decision support for sustainable nitrogen management.

### 2.1 Smart sensing for site-specific nitrogen response modeling and point EONR prescriptions

A substantial body of research has modeled crop yield response to nitrogen and derived the economically optimal nitrogen rate (EONR) by balancing expected yield gains against fertilizer costs (Ransom et al., 2023; Park et al., 2024). In plant science and PA, these efforts increasingly rely on smart sensing inputs such as weather observations, soil information, crop reflectance and georeferenced management data to estimate spatially varying crop response (Baum et al., 2023; Thompson et al., 2024). Process-based and systems modeling approaches have been used to forecast optimum nitrogen rates under variable environmental conditions, supporting more consistent season-to-season planning and making explicit the role of plant-environment interactions in nitrogen response (Baum et al., 2023; Thompson et al., 2024).

Remote sensing and on-farm experimentation have further expanded the ability to estimate subfield-scale nitrogen response and generate EONR maps (Abdipourchenarestansofla and Piepho, 2025). Piikki et al. introduce a two-step strategy integrating remote sensing with on-farm experiments to estimate optimal nitrogen rates, illustrating how scalable sensing can support spatially explicit prescriptions (Khan et al., 2026d; Piikki et al., 2022). On-farm precision experimentation has become especially valuable because it produces dense, georeferenced response data under real management conditions and strengthens the empirical basis for variable-rate recommendation (Loewen and Maxwell, 2024; Puntel et al., 2024). More broadly, these studies show that smart sensing can move nitrogen management away from uniform treatment and toward subfield-specific intervention.

However, most of this literature still treats decision making primarily as a point-estimation problem (Hegedus et al., 2023; Kakhani et al., 2024). The true site-specific EONR cannot be directly observed, so evaluation often depends on predictive accuracy or *post-hoc* comparisons that do not fully represent the decision objective (Matavel et al., 2024). de Lara et al. explicitly highlight the distinction between validating yield predictions and validating EONR estimates (Leitão et al., 2025; A Samoah et al., 2024). Nigon et al. demonstrate that uncertainty can be quantified for EONR through profile-likelihood confidence intervals under specified response functions, but these approaches still depend on strong functional assumptions and typically aim to identify a single optimum (Franc et al., 2023). As a result, within-field recommendations may remain fragile when uncertainty is high or when several nitrogen rates are economically similar. This is a critical issue for smart sensing-enabled deployment, where users require not only informed prescriptions but also trustworthy ones.

### 2.2 Uncertainty quantification, decision reliability and the gap to sustainable phytoprotection

Uncertainty quantification in agronomic prediction has been explored using probabilistic and quantilebased methods such as quantile regression forests, which generate prediction intervals or full predictive distributions (Husnain et al., 2025). These approaches help characterize variability in yield forecasts, but they generally operate at the prediction level rather than the prescription level and often lack finite-sample validity guarantees (Sousa et al., 2024). This distinction is important in nitrogen management because the operational question is not merely how uncertain yield is, but whether a recommended action remains acceptable when uncertainty, heterogeneity and downside risk are taken into account (Chen et al., 2024). From a plant science perspective, this reliability issue matters because uncertain nutrient decisions can undermine both productivity and environmentally responsible crop management.

Conformal prediction has emerged as a particularly relevant methodological advance because it can provide prediction intervals with coverage guarantees under exchangeability while remaining largely modelagnostic (Jeary et al., 2026). Romano et al. introduced conformalized quantile regression to reduce conservatism under heteroscedasticity and subsequent work has developed stronger variants for distributional and approximately conditional validity (Candès et al., 2023). Conformal methods have also shown promise in agriculture-adjacent and field settings, including classification under uncontrolled conditions and geospatial regression for environmental variables (Farag et al., 2025; Khan et al., 2026a). These developments suggest that CP is well suited to smart sensing contexts, where data are heterogeneous, field conditions are variable and calibrated reliability is essential.

Yet a gap remains between calibrated prediction and calibrated action (Haas and Hullermeier, 2025). In most agricultural applications, conformal methods are used to improve prediction reliability, but the final recommendation step still relies on point optimization. As a result, decision support may continue to hide downside risk even when uncertainty is explicitly estimated. A related gap concerns selective recommendation. In many within-field situations, especially where sensor signals are weak, conditions are atypical or competing treatments are economically indistinguishable, the most responsible action may be to abstain and request more information rather than force a prescription (Angelopoulos and Bates, 2023; Khan et al., 2026b). This capability is highly relevant to smart sensing workflows and to sustainable phytoprotection because it encourages cautious intervention when available evidence is insufficient.

**Supplementary Table 1** summarizes representative literature in smart sensing-enabled nitrogen management, highlights the key reliability gaps under uncertainty and situates the proposed uncertainty-aware, LCB-based prescription framework relative to existing work. Two major limitations persist. First, uncertainty is usually reported only for yield prediction, while the prescription stage still depends on point optimization, leaving decision reliability under plant-environment uncertainty insufficiently addressed. Second, the literature rarely incorporates selective recommendation through abstention, despite its clear operational value in field-scale decision support. When uncertainty is excessive, abstention can prompt additional sensing, further sampling, conservative defaults or targeted strip trials rather than forcing potentially unreliable intervention. The proposed framework builds on sensing-informed site-specific nitrogen management but extends it by propagating calibrated yield uncertainty into profit bounds and by selecting nitrogen rates through a risk-aware LCB rule with an explicit abstention mechanism. In this way, it contributes to more trustworthy smart sensing-driven nutrient stewardship.

## 3 Methodology

This section presents the proposed uncertainty-aware fertilizer prescription framework, including the yield-response modeling strategy, conformal uncertainty quantification procedure, profit-based uncertainty propagation and risk-aware nitrogen decision rules used to produce reliable prescriptions.

### 3.1 Problem formulation and notation

Site-specific nitrogen management is formulated as a decision problem under within-field variability, where a fertilizer recommendation must be produced from contextual covariates and a candidate nitrogen rate. Let x∈X denote a vector of contextual covariates describing local field conditions, including soil and terrain properties, weather-related indicators, crop-condition signals and management factors. Let N∈N denote the fertilizer decision variable, interpreted as an applied nitrogen-rate proxy. Yield is modeled through the response function as shown in Equation 1:

(1) $$\hat{y}=f\left(x,N\right),$$

where *f* is a supervised regression model trained to predict crop yield.

Because the objective is a reliable prescription rather than a point prediction alone, predictive uncertainty is quantified through prediction intervals with target coverage 1 − *α*, where *α* ∈ (0,1) is a user-selected miscoverage level. To connect predictive uncertainty to an operational decision objective, context-dependent economic profit is defined as shown in Equation 2:

(2) $$\pi \left(x,N\right)=p_{g}\;y\left(x,N\right)-c_{N}\;N-c_{\text{app}}-\lambda \;\phi \left(x,N\right),$$

where *p_g_* is the crop price, *c_N_* is the fertilizer unit cost, *c*_app_ is a fixed application cost, *ϕ*(*x*, *N*) is an environmental proxy capturing loss-related concerns under within-field variability and *λ* ≥ 0–0 controls the penalty strength.

### 3.2 Dataset and study variables

This study uses the *Agricultural Yield Prediction Dataset* from Kaggle, consisting of 10,000 observations and 46 variables. The target variable is crop yield, denoted as Yield and treated as a continuous response. The fertilizer decision variable is the nitrogen-rate proxy *N*, represented by the numeric column N. Contextual covariates *x* are organized into four feature groups aligned with major drivers of within-field variability and their distinct roles in prescription design.

*Weather and climate variables* include temperature, humidity, rainfall, solar radiation, wind speed and growing degree days. These variables capture short-horizon environmental forcing and are expected to be informative for immediate yield responsiveness, although they may vary substantially across samples and seasons. *Soil and terrain variables* include soil type, pH, electrical conductivity organic carbon, cation exchange capacity, sand, silt, clay fractions, bulk density, water-holding capacity, slope, aspect and elevation. These variables describe slower-varying structural field conditions and are expected to provide a relatively stable basis for prescription decisions. *Crop-condition variables* include normalized difference vegetation index (NDVI), enhanced vegetation index (EVI), leaf area index (LAI) and chlorophyll. These variables summarize in-season canopy state and plant vigor, thereby providing direct observational evidence of crop response that can sharpen site-specific recommendations. *Management and categorical identifiers* include crop type, growth stage, irrigation frequency, fertilizer type, pesticide usage, region, season and year. These variables capture intervention context and broad stratification effects that align prescriptions with agronomic setting.

This grouping is used both to structure the model inputs and to support feature-group ablations in Section 5.5, where the relative contribution of each proxy family to profitability and decision stability is assessed. Date fields are converted into numeric season-timing features using planting and harvest timestamps and all numeric variables are standardized after unit-consistency checks.

### 3.3 Data preprocessing, quality control and leakage-free splitting

#### 3.3.1 Missingness assessment and preprocessing

A standardized preprocessing pipeline is applied before model training to improve data quality and preserve evaluation validity under realistic deployment shift. Let $D={\left\{\left(x_{i},N_{i},y_{i}\right)\right\}}_{i=1}^{n}$ denote the raw dataset. Because the practical importance of imputation depends on the amount of missing data, feature-wise missingness is quantified before any preprocessing step. **Supplementary Table 2** reports the proportion of missing entries for each feature, allowing the reader to judge whether median and unknown-category imputation act primarily as minor cleanup steps or as stronger modeling assumptions for particular variables.

For each feature *z* ∈ *x*, missing values are imputed using a rule determined by feature type. Numeric features are imputed with the median computed on the training split, while categorical features are imputed with a dedicated unknown category. Outliers in numeric variables, including *y* and *N*, are clipped through quantile-based winsorization to reduce the influence of extreme values while preserving rank structure. Categorical variables are one-hot encoded and all numeric variables are standardized using training statistics only, thereby preventing leakage into calibration and test partitions. Each numeric feature *z* is transformed as shown in Equation 3:

(3) $$z_{i}^{\prime }=\frac{z_{i}-\mu_{z}^{\text{tr}}}{\sigma_{z}^{\text{tr}}+\epsilon },$$

where $\mu_{z}^{tr}$ and $\sigma_{z}^{tr}$ are the mean and standard deviation computed on the training split and *ϵ* = 10^−8^ is a fixed numerical-stability constant. Duplicate records are removed by exact row matching after type normalization of date and categorical fields.

#### 3.3.2 Group-key construction and leakage-free split design

To evaluate generalization in a manner consistent with within-field and across-season deployment, a leakage-free splitting strategy based on agronomically meaningful groups is used. For each record, a canonical group key is constructed by normalizing the grouping fields and concatenating them in a fixed order, as shown in Equation 4:

(4) $$k_{i}=\text{norm}\left({\text{Region}}_{i}\right)∥\;|\;∥\mathrm{norm}\left({\text{CropType}}_{i}\right)∥\;|\;∥\mathrm{fmt}\left({\text{Year}}_{i}\right)∥\;|\;∥\mathrm{norm}\left({\text{Season}}_{i}\right),$$

where norm(·) trims whitespace, converts strings to lowercase, maps missing categorical values to unknown and standardizes labels to a fixed vocabulary, while fmt(·) converts the year into a fixed string representation. The group identifier is then defined by applying a deterministic hash function to the UTF-8 encoding of *k_i_*,

(5) $$g_{i}=\text{SHA}256\left(\text{UTF}8\left(k_{i}\right)\right).$$

The use of a canonical key together with a platform-independent hash avoids the run-to-run instability of language-dependent hash implementations and ensures that identical grouping tuples map to the same identifier across environments.

The dataset is partitioned into training, calibration and test sets by sampling disjoint groups rather than individual rows. Specifically, the set of unique group identifiers is first sorted lexicographically and then assigned to train, calibration and test partitions using a fixed split seed 42 and target proportions 60/20/20. This produces a single grouped split that is reused for preprocessing, model fitting, model selection, conformal calibration and final evaluation unless stated otherwise. No repeated group resampling is used in the main experiments. The split therefore satisfies $G_{\text{tr}}\cap G_{\text{cal}}=\emptyset$, $G_{\text{tr}}\cap G_{\text{te}}=\emptyset$ and $G_{\text{cal}}\cap G_{\text{te}}=\emptyset$, ensuring that preprocessing statistics, model training and uncertainty calibration do not leak information across partitions. Because the group-key construction, hash function, sorting rule and random seed are fixed, the split is fully reproducible across runs and platforms.

#### 3.3.3 Split diagnostics and data-quality summary

The purpose of this grouped split is not merely to preserve similar pooled marginals across train, calibration and test sets, but to evaluate robustness under conditional drift across unseen region-cropseason combinations. Marginal distribution similarity is therefore treated only as a weak sanity check rather than a sufficient justification of split validity. The more relevant question is whether the learned prescription rule remains reliable when applied to groups whose joint covariate structure, yield-response behavior and decision characteristics were not observed during training. Accordingly, the main evidence for split adequacy comes from disjoint-group evaluation together with group-wise reliability, abstention and decision-robustness analyses reported later in the manuscript, rather than from pooled marginal comparisons alone.

**Supplementary Table 3** summarizes the preprocessing choices and split artifacts. The training-only fitting of imputers, encoders and scalers reported in **Supplementary Table 3**, together with the deterministic group construction in Equations 4, 5, is central to the reliability objective because it tests whether prescriptions remain stable when applied to unseen region, crop and season combinations.

In addition to reporting the number of samples and groups in each split, the distribution of group sizes is also summarized. **Supplementary Table 4** reports the minimum, median, mean, interquartile range and maximum group size within each partition, together with the number of very small groups. These statistics clarify whether the region-crop-season grouping is broadly balanced or dominated by a small number of large groups.

#### 3.3.4 Implications for downstream modeling and calibration

After preprocessing and group-based splitting, a consistent basis is obtained for training the yield-response model in Equation 6 and calibrating uncertainty without leakage using Equations 10, 11. This structure ensures that uncertainty estimates reflect genuine distribution shift across unseen groups, which is the relevant setting for decision support at within-field scale.

### 3.4 Yield response model training

#### 3.4.1 Problem formulation and training objective

Supervised regression models are trained to learn the yield-response function that maps contextual covariates and nitrogen rate to yield. Let $x_{i}\in {\mathbb{R}}^{d}$ denote the preprocessed feature vector for observation *i*, let *N_i_*denote the nitrogen-rate proxy and let *y_i_*denote the observed yield. Nitrogen is concatenated with the contextual features and yield is modeled as

(6) $${\hat{y}}_{i}=f_{\theta }\left(\left[x_{i},\;N_{i}\right]\right),$$

where *f_θ_* is a parametric predictor with parameters *θ*. All candidate models are trained on the training split by minimizing empirical risk under squared-error loss, as shown in Equation 7:

(7) $$\theta^{*}=\text{arg }\underset{\theta }{\min}\;\frac{1}{\left|D_{tr}\right|}\sum_{i\in D_{\text{tr}}}{\left(y_{i}-{\hat{y}}_{i}\right)}^{2},$$

and candidate models are evaluated on the calibration split using root mean squared error,

(8) $$\text{RMSE}=\sqrt{\frac{1}{\left|D\right|}\sum_{i\in D}{\left(y_{i}-{\hat{y}}_{i}\right)}^{2}}.$$

#### 3.4.2 Candidate model classes

Three model classes commonly used in agronomic prediction are considered. A linear ridge-regression baseline provides an interpretable reference and evaluates whether yield response can be captured through approximately linear effects. A generalized additive baseline provides moderate flexibility while retaining partial interpretability. Specifically, each continuous predictor is expanded using cubic B-spline basis functions with 5 knots per feature, where knot locations are placed at training-split empirical quantiles only. The additive predictor is written as shown in Equation 9:

(9) $${\hat{y}}_{i}=\beta_{0}+\sum_{j=1}^{d+1}f_{j}\left(v_{ij}\right),$$

where *v_ij_* denotes the *j*th scalar input in [*x_i_*, *N_i_*] and each *f_j_*(·) is represented by the chosen spline basis. Unless stated otherwise, the generalized additive model does not include pairwise interaction terms, so its capacity is controlled by the number of basis functions per feature and by a ridge penalty *η* = 1.0. This makes the comparator more flexible than linear regression while remaining substantially more structured than the tree-ensemble alternative.

A tree-ensemble regressor is used to capture nonlinear interactions among soil, weather, vegetation indices and management variables, which are important when within-field response is heterogeneous. The feature set includes all preprocessed numeric covariates and one-hot encoded categorical variables, with nitrogen rate explicitly included so that prescription can be performed by sweeping candidate *N* values at inference time.

#### 3.4.3 Model selection on the grouped calibration split

Model selection is performed once on the single fixed grouped calibration split defined by Equation 5. In the main setting, each candidate predictor family is trained once on D_tr_ and scored once on the held-out grouped calibration split using RMSE, after which the model with the lowest calibration RMSE is selected for all downstream experiments. To make the stability of this choice explicit, the single-split calibration RMSE values are reported directly: 1.982 for ridge regression, 1.764 for the additive baseline and 1.531 for the tree ensemble. This separation makes clear that model-selection uncertainty is distinct from conformal uncertainty and that the calibration split is used first for model selection and only afterward, once the base predictor is fixed, for conformal residual quantile estimation.

#### 3.4.4 Selected backbone and alignment of downstream comparators

Following this comparison, the tree-ensemble regressor is selected as the base yield model for the main prescription pipeline and is kept fixed for all downstream point-based comparisons. The expected-profit and point-estimate EONR baselines therefore use the same predictive backbone as the main model and differ only in the decision rule applied to its predictions.

For interval-based comparators, the same predictor family is reused whenever the uncertainty method is compatible with that learner. In particular, the ensemble baseline is instantiated using the same predictor family as the selected main model so that the comparison isolates the effect of interval construction rather than a change in predictive backbone. The dropout baseline uses a dedicated multilayer perceptron regressor with dropout layers because Monte Carlo dropout requires stochastic dropout layers to remain active at test time and the selected main model is non-neural. This design choice is stated explicitly so that any unavoidable learner mismatch remains transparent.

### 3.5 Conformal prediction for yield intervals

To quantify predictive uncertainty with finite-sample guarantees, split conformal prediction (CP) is applied on top of the trained yield-response model in Equation 6. After fitting $f_{\theta^{*}}$ on the training split, absolute residualscores are computed on the disjoint calibration split $D_{\text{cal}}$ as shown in Equation 10:

(10) $$s_{i}=\left|y_{i}-{\hat{y}}_{i}\right|,\;{\hat{y}}_{i}=f_{\theta^{*}}\;\left(\left[x_{i},\;N_{i}\right]\right),\;i\in D_{\text{cal}}.$$

For a desired miscoverage level α∈(0,1), the conformal quantile $q_{1-\alpha }$ is obtained as the empirical (1−*α*) quantile of ${\left\{s_{i}\right\}}_{i\in D_{\text{cal}}}$ using the standard finite-sample correction,

(11) $$q_{1-\alpha }=\text{Quantile}\left(\left\{s_{i}\right\},\;\left(\left|D_{\text{cal}}\right|+1\right)\left(1-\alpha \right)/\left|D_{\text{cal}}\right|\right).$$

Given a test point (*x*, *N*), a two-sided prediction interval is produced by symmetrically expanding the point estimate,

(12) $${\hat{C}}_{1-\alpha }\left(x,N\right)=\left[\hat{y}\left(x,N\right)-q_{1-\alpha },\;\hat{y}\left(x,N\right)+q_{1-\alpha }\right].$$

Results are reported at coverage levels 1−*α* ∈ {0.90,0.95} because these settings are practically relevant for risk-sensitive fertilizer decisions. Under the exchangeability assumption between calibration and test samples, the interval in Equation 12 achieves marginal coverage at or above the target level, thereby providing a principled basis for downstream economic decision rules. **Supplementary Figure 4** illustrates how finite-sample uncertainty is constructed and propagated through the split conformal procedure.

### 3.6 Profit modeling and interval propagation

To translate predictive uncertainty into decision-relevant uncertainty, an economic profit model is defined for each candidate nitrogen rate *N* under context *x*. Profit is written explicitly as

π(x,N) to emphasize that the objective depends jointly on contextual covariates and the candidate nitrogen decision. The profit model is

(13) $$\pi \left(x,N\right)=p_{g}\;y\left(x,N\right)-c_{N}\;N-c_{\text{app}}-\lambda \;\phi \left(x,N\right),$$

where *p_g_* is the grain price per yield unit, *c_N_* is the nitrogen unit cost, *c*_app_ is a fixed application cost and *λ* ≥ 0 controls the strength of an environmental penalty. The term ϕ(x,N) is an environmental proxy representing loss-related concerns under within-field variability. In this study, the surplus proxy is defined as shown in Equation 14:

(14) $$\phi \left(x,N\right)=\text{max }\left(0,\;N-\tilde{N}\left(x\right)\right),$$

where $\tilde{N}\left(x\right)$ is the context-dependent reference nitrogen rate used to define surplus. To make this proxy fully reproducible, the reference rate is defined operationally as shown in Equation 15:

(15) $$\tilde{N}\left(x\right)=\{\begin{array}{ll} \frac{1}{\left|D_{tr}^{\left(r,c\right)}\right|}\sum_{i\in D_{\text{tr}}^{\left(r,c\right)}}N_{i}, & \text{if}\;\left|D_{\text{tr}}^{\left(r,c\right)}\right|>0,\\ 105.19, & \text{otherwise}, \end{array}$$

where *r* = Region(*x*), *c* = CropType(*x*) in Equation 16:

(16) $$D_{\text{tr}}^{\left(r,c\right)}=\left\{i\in D_{\text{tr}}:{\text{Region}}_{i}=r,\;{\text{CropType}}_{i}=c\right\}.$$

Under this definition, $\tilde{N}\left(x\right)$ is computed deterministically from the available context using the training-split mean nitrogen rate for the corresponding crop-region combination and is then held fixed when evaluating candidate rates *N*. If a crop-region subgroup is absent in the training split, the global reference rate 105.19 is used. The penalty therefore activates only when the prescribed nitrogen exceeds the context-specific reference level, making ϕ(x,N) exactly reproducible once $\tilde{N}\left(x\right)$ is specified.

Given the conformal yield prediction interval ${\hat{C}}_{1-\alpha }\left(x,N\right)=\left[L_{y}\left(x,N\right),U_{y}\left(x,N\right)\right]$ from Equation 12, uncertainty is propagated to the economic objective by applying Equation 13 to the interval endpoints,

(17) $${\hat{I}}_{1-\alpha }^{\pi }\left(x,N\right)=\left[p_{g}\;L_{y}\left(x,N\right)-c_{N}\;N-c_{\text{app}}-\lambda \;\phi \left(x,N\right),p_{g}\;U_{y}\left(x,N\right)-c_{N}\;N-c_{\text{app}}-\lambda \;\phi \left(x,N\right)\right].$$

This mapping yields conservative profit bounds when uncertainty in yield dominates uncertainty in costs, which is consistent with the focus of this study on uncertainty-aware prescriptions. The baseline economic parameters used in Equation 13 are listed in **Supplementary Table 5**. These values define the reference operating condition for the main experiments, while the sensitivity analysis in Section 4.3 perturbs them under alternative economic scenarios.

### 3.7 Risk-aware fertilizer prescription rule

For each context *x*, prescriptions are generated by evaluating a discrete grid of candidate nitrogen rates,

(18) $$N_{\text{grid}}=\left\{N_{\text{min }},\;N_{\text{min }}+\text{Δ}N,\;\ldots ,\;N_{\text{max }}\right\},$$

where in this study *N*_min_ = 10 kg*/*ha, *N*_max_ = 200 kg*/*ha and ΔN=5 kg/ha. For each candidate $N\in N_{\text{grid}}$, a profit interval ${\hat{I}}_{1-\alpha }^{\pi }\left(x,N\right)=\left[L_{\pi }\left(x,N\right),U_{\pi }\left(x,N\right)\right]$ is obtained from Equation 17. The lower confidence bound for context-specific profit is defined as shown in Equation 19:

(19) $${\text{LCB}}_{1-\alpha }\;\left(\pi \left(x,N\right)\right)=L_{\pi }\left(x,N\right),$$

and the risk-aware prescription is selected by maximizing this lower bound,

(20) $$N_{\text{LCB}}^{*}\left(x\right)=\text{arg }\underset{N\in N_{\text{grid}}}{\max}\;{\text{LCB}}_{1-\alpha }\;\left(\pi \left(x,N\right)\right).$$

This criterion favors nitrogen rates that maximize a conservative profit guarantee for the given context *x* under miscoverage level *α*, thereby directly targeting decision reliability under uncertainty.

As a comparator, an expected-profit prescription is computed by using the point yield estimate $\hat{y}\left(x,N\right)$ in the same context-dependent profit model and selecting the maximizing rate,

(21) $$N_{E}^{*}\left(x\right)=\text{arg }\underset{N\in N_{\text{grid}}}{\max}\;\hat{\pi }\left(x,N\right),$$

where in Equation 22:

(22) $$\hat{\pi }\left(x,N\right)=p_{g}\;\hat{y}\left(x,N\right)-c_{N}\;N-c_{\text{app}}-\lambda \;\phi \left(x,N\right).$$

The contrast between the risk-aware rule in Equation 20 and the expected-profit rule in Equation 21 isolates the effect of uncertainty calibration and conservative optimization on site-specific nitrogen prescriptions under within-field variability. In both cases, the optimization objective is written explicitly as a function of (*x,N*) so that contextual dependence remains visible throughout the decision formulation.

The risk-aware prescription mechanism is illustrated in **Supplementary Figure 5**, showing how conservative optimization over context-dependent profit lower bounds produces stable nitrogen decisions and supports abstention under low confidence.

### 3.8 End-to-end framework for risk-aware and selective nitrogen prescription

**Supplementary Figure 6** provides an end-to-end overview of the proposed pipeline, illustrating how heterogeneous agronomic data are transformed into reliable, uncertainty-aware nitrogen prescriptions. The framework integrates leakage-free evaluation, predictive uncertainty quantification, profit propagation and selective decision-making into a single deployment-oriented workflow.

The pipeline begins with a heterogeneous agronomic dataset comprising yield targets, nitrogen rates, weather variables, soil and terrain attributes, vegetation indices and management identifiers. Preprocessing addresses missing values, outliers, duplicates and unit inconsistencies, after which the data are partitioned using a group-based split over region, crop, season and year, following the leakage-free protocol summarized in **Supplementary Table 3**. The yield-response model f(x,N) in Equation 6 maps contextual features and candidate nitrogen rates to expected yield. A tree-based ensemble is selected to capture nonlinear and heterogeneous responses across deployment contexts. Predictive uncertainty is introduced through split conformal calibration, producing finite-sample valid yield intervals as defined in Equation 11 and evaluated in **Supplementary Table 12** and **Supplementary Figure 10**. Yield intervals are then propagated through the profit model in Equation 13, enabling lower-confidence-bound optimization via Equation 20. Selective recommendation abstains under high uncertainty, thereby linking uncertainty quantification to reliable agronomic decisions, as reported in **Supplementary Table 16** and **Supplementary Figures S13**, **S14**.

## 4 Experimental setup

This section defines the evaluation protocol, baseline methods, ablation design and economic sensitivity scenarios used to assess the proposed risk-aware prescription framework in a fair and reproducible manner.

### 4.1 Baselines and comparators

To determine whether uncertainty-aware prescriptions improve decision reliability under within-field variability, the proposed pipeline is compared against baselines representing common agronomic practice and representative uncertainty-estimation strategies.

#### 4.1.1 Point-based baselines

To determine whether uncertainty-aware prescriptions improve decision reliability under within-field variability, the proposed pipeline is compared against point-based baselines that reflect common agronomic practice and standard prescription rules without explicit uncertainty handling.

The *uniform fertilizer* baseline applies a constant nitrogen rate to all samples, set to the training-split mean of *N* and represents non-site-specific management. The *expected profit* baseline implements the point-optimization rule in Equation 21 and selects the nitrogen rate that maximizes point-estimated profit. The *point estimate EONR* baseline computes the profit-maximizing rate on the candidate grid using the same yield-response model as the main pipeline but without uncertainty bounds and treats the resulting rate as a point recommendation.

These point-based comparators therefore differ from the proposed method only in decision policy, not in the underlying predictor family. This design directly addresses the gap highlighted in Section 2, where prescriptions are commonly derived from point predictions even when predictive uncertainty is decision-relevant. The candidate yield-model families from which the base predictor is selected are defined in Section 3.4, including the generalized additive baseline with cubic B-spline basis functions, 5 knots per feature and ridge regularization with *η* = 1.0, so that the effective capacity of each learner remains transparent.

#### 4.1.2 Uncertainty-oriented baselines

Uncertainty-oriented comparators are also included. These methods construct predictive intervals but do not provide the same finite-sample coverage guarantees as conformal prediction.

The *quantile regression* baseline estimates lower and upper conditional quantiles of yield for each [*x,N*] pair and forms intervals directly from these predicted quantiles using *α/*2 and 1 − *α/*2. Accordingly, the interval endpoints correspond to (0.05,0.95) for 1 − *α* = 0.90 and (0.025,0.975) for 1 − *α* = 0.95.

The *ensemble* baseline uses the same predictor family as the selected main model, namely the treeensemble regressor and trains *M* = 10 independently fitted members on the same training split, with diversity induced through bootstrap resampling. For each candidate [*x,N*], the predictive interval is obtained from the empirical distribution of the *M* model outputs using a central (1−*α*) empirical percentile interval.

The *dropout* baseline uses a dedicated multilayer perceptron regressor with dropout layers and approximates predictive uncertainty through Monte Carlo dropout. Dropout with rate *p* = 0.20 is applied after each hidden layer and *T* = 50 stochastic test-time forward passes are performed for each candidate input. The resulting predictive interval is constructed from the sampled predictions using a percentile interval. The underlying learner is stated explicitly because Monte Carlo dropout is only defined for models with stochastic dropout layers and therefore does not share the exact same predictor class as the main conformal model in this study, whose selected backbone is non-neural.

#### 4.1.3 Shared evaluation protocol and fairness of comparison

Whenever these methods produce yield intervals, the intervals are mapped to profit intervals through the same profit model in Equation 13, ensuring that all uncertainty-aware baselines are evaluated under an identical downstream economic decision layer. **Supplementary Table 6** summarizes the full set of evaluated methods.

All baselines share identical group splits, nitrogen grids in Equation 18 and economic parameters in **Supplementary Table 5**, so that performance differences arise from the uncertainty-estimation and decision-policy mechanisms rather than from changes in data partitioning or economic assumptions.

### 4.2 Ablation studies

Targeted ablation studies are used to quantify how individual components contribute to the reliability objectives introduced in Section 3. Each ablation removes or alters a single component while keeping the remaining pipeline unchanged, including the group-split protocol in Equation 5, the nitrogen grid in Equation 18 and the interval-to-profit mapping in Equation 17.

First, the environmental penalty is removed by setting *λ* = 0 in Equation 13, thereby testing whether sustainability-aligned regularization changes prescriptions and outcome stability. Second, selective recommendation is removed by disabling abstention and forcing a prescription for every sample, which isolates the operational value of explicitly acknowledging low-confidence cases. Third, feature groups are ablated to examine how different measurement sources support site-specific decisions. In this setting, models are trained using weather and climate variables only, soil and terrain variables only, crop-condition indices only and the full feature set, after which yield accuracy, interval calibration and prescription stability are compared across these variants.

The effect of uncertainty stringency is also examined by comparing conformal coverage levels 1 − *α* ∈ {0.90,0.95}. This changes the quantile in Equation 11 and therefore the conservatism of the lower confidence bound used in Equation 20. **Supplementary Table 7** summarizes the ablation configurations used in experiments.

### 4.3 Sensitivity analysis

Because fertilizer decisions are jointly shaped by economic and environmental factors, robustness to parameter variability is assessed by varying grain price *p_g_*, nitrogen cost *c_N_*, application cost *c*_app_ and environmental penalty weight *λ* in the profit model of Equation 13. Let S denote the set of economic scenarios.

For each scenario *s* ∈ S with parameters $\left(p_{g}^{\left(s\right)},c_{N}^{\left(s\right)},c_{\text{app}}^{\left(s\right)},\lambda^{\left(s\right)}\right)$, profit is recomputed as shown in Equation 23:

(23) $$\pi^{\left(s\right)}\left(x,N\right)=p_{g}^{\left(s\right)}\;y\left(x,N\right)-c_{N}^{\left(s\right)}\;N-c_{\text{app}}^{\left(s\right)}-\lambda^{\left(s\right)}\;\phi \left(x,N\right),$$

and scenario-specific prescriptions are obtained through the same risk-aware decision rule in Equation 24,

(24) $$N_{\text{LCB}}^{*,\left(s\right)}\left(x\right)=\text{arg }\underset{N\in N_{\text{grid}}}{\max}\;{\text{LCB}}_{1-\alpha }\;\left(\pi^{\left(s\right)}\left(x,N\right)\right).$$

Prescription sensitivity is quantified by comparing the distribution of $N_{\text{LCB}}^{*,\left(s\right)}$ across scenarios and reporting both the mean absolute change in prescribed rate and the fraction of samples whose prescription changes beyond a practical threshold. A test sample is counted as having a practically meaningful shift as below in Equation 25:

(25) $$\left|N_{\text{LCB}}^{*,\left(s\right)}\left(x\right)-N_{\text{LCB}}^{*,\text{base}}\left(x\right)\right|>{\text{Δ}}_{N}^{\text{prac}},$$

where ${\text{Δ}}_{N}^{\text{prac}}=5\;\text{kg}/\text{ha}$ denotes the minimum change considered operationally meaningful. This threshold reflects practical application granularity and a conservative minimum prescription shift that would matter in field deployment.

To ensure that observed changes reflect economic sensitivity rather than changes in predictive components, all scenarios reuse the same trained yield model in Equation 6, the same conformal quantiles derived from Equations 10, 11 and the same nitrogen grid in Equation 18. **Supplementary Table 8** summarizes the scenario design used throughout experiments. Explicitly listing the values of *p_g_*, *c_N_*, *c*_app_ and *λ* makes the perturbation design fully reproducible and clarifies how each scenario deviates from the baseline operating condition in **Supplementary Table 5**. Each scenario specifies explicit values of grain price, nitrogen cost, application cost and environmental penalty weight. A prescription is counted as practically changed when $\left|N_{\text{LCB}}^{*,\left(s\right)}-N_{\text{LCB}}^{*,\text{base}}\right|>{\text{Δ}}_{N}^{\text{prac}}$, with ${\text{Δ}}_{N}^{\text{prac}}=5\;\text{kg}/\text{ha}$.

## 5 Results

This section presents empirical evidence evaluating the proposed risk-aware nitrogen prescription framework under realistic, leakage-free deployment settings. We analyze data heterogeneity, uncertainty calibration, decision robustness and selective recommendation behavior to demonstrate how uncertaintyaware optimization improves reliability relative to point-based baselines.

### 5.1 Data heterogeneity motivating risk-aware prescriptions

The proposed framework targets settings characterized by substantial within-field and across-season variability. The analysis therefore begins by quantifying the dispersion of key agronomic drivers entering the yield-response model *f*(*x,N*) in Equation 6 and the profit function *π*(*x,N*) in Equation 13. Large variability in both management intensity and environmental covariates suggests that a single uniform rule is unlikely to perform reliably across contexts, thereby motivating uncertainty-aware prescriptions beyond point EONR.

**Supplementary Table 9** summarizes descriptive statistics after preprocessing. The broad spread in yield, nitrogen rate, weather variables, soil properties and crop-condition indices provides practical support for flexible nonlinear modeling and for explicit propagation of predictive uncertainty into the decision layer through Equation 20.

**Supplementary Figure 7** shows that most pairwise correlations among numerical predictors are modest, indicating the absence of severe multicollinearity across soil, weather, management and crop-index variables. This supports stable use of flexible nonlinear models for *f*(*x,N*), such as tree-based ensembles and reduces the risk that redundant predictors inflate estimation noise. As a result, uncertainty propagated to profit bounds through Equation 17 is more likely to reflect intrinsic agronomic variability, thereby strengthening the reliability of the LCB prescription rule in Equation 20.

To connect heterogeneity more directly to the decision objective, yield-response patterns are visualized across deployment-relevant strata. **Supplementary Figure 8** shows that yield-*N* relationships vary by region in both trend and dispersion, indicating that a global point EONR is an unreliable proxy for context-specific prescriptions. This observation further motivates evaluation under a split protocol that reflects realistic generalization.

### 5.2 Leakage-free evaluation and yield model selection

The heterogeneity observed in **Supplementary Table 9** and **Supplementary Figure 8** implies that evaluation should reflect deploymentlike shifts rather than random i.i.d. partitions. Results are therefore reported under the leakage-free group-splitting protocol defined in Equation 5. **Supplementary Table 10** summarizes the resulting partition and confirms disjoint groups across training, calibration and test splits.

To support fair comparison, the grouped split is also checked for large accidental shifts in key decision variables. **Supplementary Figure 9** presents split-wise empirical distributions of nitrogen and yield. The close alignment across splits indicates that downstream performance differences are less likely to be artifacts of marginal distribution mismatch and more likely to reflect method behavior under group generalization.

Given the evaluation protocol in **Supplementary Table 10**, the next step is selection of the yield model used for conformal calibration in Equation 11 and for decision propagation in Equation 17. **Supplementary Table 11** reports calibration-split predictive accuracy. The tree ensemble achieves the lowest RMSE and MAE and is therefore selected as the base predictor for subsequent uncertainty and decision experiments.

### 5.3 Conformal calibration and group reliability

Using the base predictor selected in **Supplementary Table 11**, conformal calibration is assessed under the leakage-free protocol. Reliable uncertainty is necessary to support profit bounds in Equation 17 and the LCB rule in Equation 20. As shown in **Supplementary Table 12**, empirical coverage on the test split closely matches the nominal levels, 1 − *α* ∈ {0.90,0.95}, supporting the use of conformal bounds for decision-making.

**Supplementary Figure 10** visualizes the same comparison between nominal and achieved coverage, highlighting that the calibrated intervals satisfy the desired reliability property under the group split in **Supplementary Table 10**. This property is essential because the LCB rule in Equation 20 operates on conservative bounds rather than on point predictions alone.

Beyond global averages, deployment requires reliability across subpopulations defined by region, crop type and season-year. **Supplementary Figure 11** reports empirical coverage by grouping at the 0.95 target.

**Supplementary Table 13** summarizes mean and worst-group behavior across both 0.90 and 0.95 targets. Together, **Supplementary Figure 11** and **Supplementary Table 13** show that coverage varies across groups and identify the most challenging subpopulations.

To connect reliability to practical operating regimes, interval width is examined against difficulty drivers such as extreme *N*, extreme weather and low vegetation index. **Supplementary Figure 12** shows both scatter and binned summaries. Wider intervals concentrate in more difficult regimes, providing a mechanistic explanation for where conservative decision rules and abstention are most likely to activate.

### 5.4 Decision performance and selective recommendation

#### 5.4.1 Uncertainty quality and resulting decision outcomes

To consolidate uncertainty quality and decision consequences into a single comparison across uncertainty baselines, **Supplementary Table 14** reports interval-quality metrics, including coverage and width statistics, together with the resulting decision performance after mapping uncertainty to profit and applying the LCB decision rule. This table links the calibration results in **Supplementary Table 12** to the prescription outcomes in **Supplementary Table 15** through Equations 17, 20.

#### 5.4.2 Aggregate decision performance

Having established reliable uncertainty under the leakage-free split in **Supplementary Table 10** and summarized group robustness in **Supplementary Figure 11** and **Supplementary Table 13**, attention now turns to the downstream decision layer. Economic parameters in Equation 13 are held fixed across all methods for fair comparison and are listed in **Supplementary Table 5**. This isolates differences in decision performance to the decision rule and uncertainty handling rather than to changes in economic assumptions.

**Supplementary Table 15** compares decision outcomes across prescription strategies on the test split. The uniform-rate baseline yields a mean profit of 4.12 with profit standard deviation 0.88, while applying a constant mean nitrogen rate of 105.19 with standard deviation 0.00, illustrating economic inefficiency despite stability.

Point EONR increases mean profit to 4.61 but with higher variability, yielding a profit standard deviation of 1.24 and substantial dispersion in nitrogen application, with mean *N* = 124.03 and standard deviation 21.57, indicating sensitivity to prediction error. The expected-profit rule achieves the highest mean profit, 4.68, with profit standard deviation 1.19 and mean *N* = 121.34 with standard deviation 18.76, but still exhibits considerable variability in both outcomes and prescriptions. In contrast, the risk-aware LCB rule attains a mean profit of 4.54 while reducing profit standard deviation to 0.96 and lowering nitrogen dispersion, with mean *N* = 112.07 and standard deviation 12.41. This reflects a modest reduction in average profit in exchange for improved stability in economic returns and input use, supporting more consistent and risk-controlled nitrogen prescriptions.

#### 5.4.3 Selective recommendation and the reliability-coverage trade-off

Selective recommendation serves as an operational mechanism that abstains when uncertainty is high, thereby aligning the decision process with the reliability objective introduced earlier. **Supplementary Figure 13** summarizes the reliability-coverage trade-off under abstention, while **Supplementary Table 16** reports abstention rates at two conformal targets. Together, these results show that increasing target coverage makes the prescription rule more conservative by withholding more recommendations.

This behavior follows directly from the conformal construction. A higher target coverage requires a larger conformal quantile *q*_1−_*_α_*on the calibration split, as defined in Equation 11. The larger quantile widens the prediction interval through Equation 12. After propagation through the profit model in Equation 17, the wider yield interval produces a wider profit interval, making candidate nitrogen rates more likely to overlap economically or fail the required downside-risk criterion in Equation 20. The decision rule therefore abstains more often at higher coverage targets.

**Supplementary Table 16** quantifies this trend. Increasing target coverage from 0.90 to 0.95 raises the abstention rate from 18.4% to 27.9% and reduces the prescribed fraction from 81.6% to 72.1%. This increase is not merely an empirical pattern but a structural consequence of the interval construction. Moving to a higher nominal coverage enlarges *q*_1−_*_α_*, which symmetrically expands the lower and upper yield bounds and therefore widens the downstream profit interval. Wider profit intervals make it more difficult to distinguish competing nitrogen rates with sufficient confidence, especially for samples with noisy covariates, weak response gradients or overlapping economic outcomes. In such cases, the framework withholds the recommendation rather than forcing a potentially fragile prescription.

This behavior is desirable in a risk-aware setting because abstention remains explicitly linked to the chosen reliability level. Lower target coverage yields a smaller *q*_1−_*_α_*, narrower intervals and broader prescription availability, but it also weakens the coverage guarantee and exposes decisions to greater downside uncertainty. Higher target coverage yields a larger *q*_1−_*_α_*, stronger uncertainty protection and greater robustness, but at the cost of fewer actionable recommendations. The abstention rates in **Supplementary Table 16** therefore provide a transparent operational summary of how conformal width inflation translates into decision conservatism, giving practitioners a tunable control for balancing reliability, usability and environmental risk.

#### 5.4.4 Abstention patterns across covariate space

To make abstention behavior more interpretable, its concentration in covariate space is visualized directly. **Supplementary Figure 14** shows abstention rates across NDVI and rainfall bins on the test split. NDVI is discretized using quantile-based bins with breakpoints [-0.85, -0.57, -0.36, -0.20, -0.07, 0.08, 0.22, 0.38, 0.59, 0.86] and rainfall is discretized using quantile-based bins with breakpoints [17.4, 55.2, 89.9, 117.1, 140.8, 161.1, 182.5, 208.0, 239.4, 328.7]. The heatmap indicates that recommendations are withheld more frequently under low-vegetation conditions and under more extreme rainfall regimes. This pattern is consistent with the interval-width increases observed in **Supplementary Figure 12**, suggesting that abstention concentrates in regions of covariate space where predictive uncertainty and economic ambiguity are highest.

#### 5.4.5 Group-wise decision robustness

To summarize robustness across deployment-relevant strata, **Supplementary Table 17** reports group-wise outcomes for both aggregate performance and paired per-sample differences relative to the expected-profit baseline. For each test sample within a group, the paired profit difference is defined as $\text{Δ}\pi_{i}=\pi_{i}^{\text{prop}}-\pi_{i}^{\text{ref}}$ and the paired prescription difference as $\text{Δ}N_{i}=N_{i}^{\text{prop}}-N_{i}^{\text{ref}}$, after which these quantities are summarized at group level. The table also reports the fraction of samples for which the proposed decision achieves higher profit than the reference rule, together with the subgroup sample size *n* contributing to each estimate. Reporting *n* helps distinguish broad, well-supported patterns from summaries driven by smaller subgroups. Interpreted together with the reliability results in **Supplementary Table 13**, these findings indicate whether stability gains are consistent across regions and crop types rather than being driven by a narrow subset of contexts.

### 5.5 Robustness: ablations and sensitivity to economic scenarios

#### 5.5.1 Component ablations and contribution of design choices

The previous subsection established decision stability under fixed economic assumptions in **Supplementary Table 5** and showed that abstention concentrates in difficult regimes in **Supplementary Figure 14**. The analysis now turns to the contribution of individual design components and to robustness under economic variability. **Supplementary Table 18** reports both ablation results and sensitivity to conformal target coverage.

Removing the environmental penalty term in Equation 13 increases mean profit from 4.54 to 4.71, but this gain is accompanied by substantially higher prescription dispersion: the standard deviation of recommended nitrogen rates increases from 12.41 to 17.95 and the mean recommended rate rises from 112.07 to 118.62. This pattern indicates that the penalty term sacrifices a modest amount of average profit in exchange for more stable and environmentally conservative prescriptions.

Disabling abstention likewise increases dispersion in both outcomes and recommendations, supporting selective recommendation as an operational reliability mechanism rather than as a cosmetic rejection option. To distinguish whether this increase reflects only a narrow tail subset or a broader spread across the decision distribution, **Supplementary Table 18** also reports interquartile-range and upper-tail summaries for profit and prescription variability. In the present estimates, the increase is broad-based: both the IQR and the P95 rise under no abstention for realized profit and recommended nitrogen rate, indicating that dispersion widens across much of the distribution rather than only in the extreme tail.

Feature-group ablations further show that different sensing proxies contribute differently to profitability and stability rather than merely shifting performance uniformly. Among the single-group variants, the *soil and terrain only* model achieves the highest mean profit (4.21) and the lowest nitrogen dispersion (18.03), indicating that structural field properties provide the strongest standalone support for stable prescriptions. By contrast, the *weather only* model yields the lowest mean profit (3.98) and the largest prescription dispersion (19.88), suggesting that short-term meteorological forcing is informative but too volatile to anchor reliable decisions on its own. The *crop indices only* model shows intermediate behavior, with mean profit 4.09 and nitrogen dispersion 18.76, implying that canopy-state proxies provide useful site-specific response information but do not by themselves deliver the same stability as slower-varying soil and terrain context. Taken together, these ablations indicate that soil and terrain proxies contribute most strongly to prescription stability, whereas crop-condition and weather signals contribute responsiveness and local adaptation. The full model performs best because it combines stable structural context with dynamic in-season sensing, allowing profitability gains without loss of control over dispersion.

#### 5.5.2 Sensitivity to conformal target coverage

Increasing the target coverage level produces more conservative behavior and higher abstention, consistent with the wider intervals reported in **Supplementary Table 12**. Lowering coverage reduces abstention and slightly increases mean profit, but at the cost of higher variability in realized profit and nitrogen prescriptions.

Taken together, **Supplementary Figure 14** and **Supplementary Tables S17, S18** indicate that the proposed uncertainty-aware prescription framework improves decision reliability without sacrificing practical usefulness. Abstention is not uniformly distributed but concentrates in regimes characterized by low vegetation signal or adverse rainfall conditions, indicating that recommendations are withheld precisely where uncertainty and downside risk are highest. Group-wise analysis shows that gains in stability are broadly consistent across regions and crop types rather than being driven by a narrow subset of contexts. Ablation results attribute these effects to key design components: removing the environmental penalty or abstention mechanism increases dispersion in both profit and nitrogen rates, while feature-group comparisons show that soil and terrain proxies contribute most strongly to prescription stability, whereas weather and crop-condition proxies contribute adaptive response information that is useful for profitability but insufficient on their own to control dispersion. Increasing conformal coverage leads to more conservative prescriptions and higher abstention, consistent with wider calibrated intervals. Sensitivity analysis under alternative economic scenarios further shows that prescriptions shift in expected directions with changes in grain price, fertilizer cost and environmental weighting while maintaining comparatively stable dispersion. Collectively, these findings indicate that reliability gains arise from calibrated uncertainty propagation, stable structural sensing context and conservative decision rules rather than from overfitting to specific conditions, supporting the framework as a robust operational approach for site-specific nitrogen management under uncertainty.

## 6 Discussion and comparison

**Supplementary Table 19** compares the proposed selective, risk-aware prescription framework with representative prior work, highlighting both the reported contributions and the aspects that are typically missing for decision reliability.

### 6.1 Uncertainty-calibrated prediction supports risk-aware nitrogen decisions

The results show that uncertainty calibration is not merely a reporting add-on, but a central mechanism that changes how nitrogen recommendations should be made. The prediction stage provides the foundation for downstream decisions and model quality therefore remains important. In the calibration comparison, the tree ensemble achieves the lowest predictive error among the candidate models, with RMSE 1.531 and MAE 1.214, indicating improved fidelity relative to the linear and additive baselines. Building on this base predictor, conformal prediction produces yield intervals whose empirical coverage closely tracks the nominal targets, including 0.912 for a 0.90 target and 0.963 for a 0.95 target, together with the expected widening of intervals as the desired reliability level increases.

This behavior matters because the decision problem is fundamentally profit-based rather than error-based. Once calibrated uncertainty is available, yield intervals can be propagated into profit intervals and used directly for decision-making. The lower-confidence-bound prescription rule formalizes a cautious decision principle in which recommendations are selected not only for high expected return, but also for protected performance under uncertainty. In this sense, calibrated prediction is valuable not simply because it improves uncertainty reporting, but because it enables a more defensible and operationally meaningful prescription rule.

### 6.2 Profit, stability and abstention define the operational trade space

The comparison among decision strategies reveals a consistent operational pattern. Expected-profit optimization tends to recommend higher nitrogen rates and achieves the highest mean profit, whereas riskaware decisions reduce nitrogen dispersion and downside variability. On the test split, the expected-profit rule achieves mean profit 4.68 with mean nitrogen 121.34 and nitrogen standard deviation 18.76, whereas the risk-aware lower-confidence-bound strategy yields mean profit 4.54 with mean nitrogen 112.07 and nitrogen standard deviation 12.41. This reduction in variability is practically important because agronomic prescriptions must be sufficiently stable to justify and deploy under real field conditions.

Selective recommendation extends this risk control by allowing abstention in high-uncertainty regions. **Supplementary Table 16** shows that the abstention rate increases from 18.4% at target coverage 0.90 to 27.9% at target coverage 0.95, reflecting increasingly conservative behavior as the decision guarantee is strengthened. The associated trade-off is explicit: stronger guarantees reduce profit volatility and stabilize prescriptions, but they also reduce the fraction of sites that receive a recommendation. The framework therefore shifts from completeness toward reliability as the desired protection level increases. This trade-off is operationally useful because it makes the balance between actionability and robustness transparent and tunable.

### 6.3 Robustness and component contributions under ablation and economic stress tests

The ablation results clarify which components are responsible for the observed reliability gains. The full model yields mean profit 4.54 with profit standard deviation 0.96 and nitrogen standard deviation 12.41, as summarized in **Supplementary Table 18**. Removing the environmental penalty increases mean profit to 4.71 but also increases variability, with profit standard deviation rising to 1.18 and nitrogen standard deviation rising to 17.95. This indicates that the sustainability-aligned penalty term contributes materially to stabilizing recommendations rather than serving as a superficial regularizer.

Disabling abstention likewise increases dispersion in outcomes and prescriptions, supporting selective recommendation as an operational reliability mechanism rather than as a cosmetic rejection option. Featuregroup ablations lead to lower performance and greater instability, showing that robust prescriptions depend on integrating complementary information sources rather than relying on weather-only or soil-only signals. The conformal coverage comparison reinforces the same theme: moving from 0.90 to 0.95 coverage reduces mean profit from 4.61 to 4.54 while lowering dispersion and increasing abstention, reflecting a systematic shift toward more conservative and reliable prescriptions as the guarantee is tightened, consistent with **Supplementary Table 16**.

Taken together, these findings show that reliability emerges from the interaction of calibrated uncertainty, conservative economic decision rules, selective abstention and context-rich sensing information. The framework therefore differs from prior point-based prescription strategies not only in predictive form, but in its explicit treatment of uncertainty as part of the decision mechanism itself.

## 7 Conclusion and future work

This study shows that smart sensing-enabled nitrogen recommendation becomes more dependable when uncertainty calibration is treated as a core component of the decision pipeline rather than as a *post-hoc* diagnostic. In precision agriculture and plant-environment management, sensing-driven recommendations must remain trustworthy under within-field heterogeneity and seasonal variability if they are to support sustainable intervention at scale. The results show that the yield-response learner provides accurate predictions and that split conformal calibration produces prediction intervals whose empirical coverage closely tracks nominal targets under leakage-free group evaluation. By propagating these calibrated yield intervals through transparent economic parameters and an environmental proxy penalty, the framework constructs profit bounds that support nitrogen prescriptions aligned with deployment-relevant goals, including protection against downside outcomes, improved nutrient-use accountability and reduced risk of environmentally harmful over-application.

Across decision strategies, a consistent operational pattern emerges. Expected-profit optimization tends to recommend higher nitrogen rates and produces larger dispersion, whereas the risk-aware lower-confidencebound rule maintains competitive mean profit with meaningfully lower variability in both prescribed nitrogen and realized profit. This is important for smart sensing applications in plant science because reliable intervention depends not only on predictive accuracy, but also on the stability and trustworthiness of recommendations derived from sensed plant and environmental variability. The selective recommendation mechanism further strengthens this reliability by abstaining when uncertainty is excessive or when candidate rates are economically indistinguishable under uncertainty. In operational terms, this creates an explicit reliability-versus-coverage trade-off that can be tuned to agronomic risk tolerance, management objectives and field-level constraints. Ablation results indicate that stability gains arise from the combined effect of calibrated uncertainty, the environmental penalty term and richer contextual information, while economic sensitivity analysis shows that recommendations adapt in the expected direction under changes in grain price, fertilizer cost and environmental weighting without destabilizing overall behavior.

Several practical directions remain for future work. First, the calibration module can be extended to better represent spatiotemporal plant-environment dynamics through online or sequential conformal updates, neighborhood-aware grouping and shift-aware calibration strategies, while preserving valid coverage under changing field conditions. Second, the decision layer can move beyond a single environmental proxy penalty toward multi-objective smart sensing-based management, incorporating explicit nutrient-loss constraints, regulatory thresholds, resource-efficiency targets or policy-aligned safeguards while retaining the profit-bound optimization principle. Third, broader validation across additional crops, regions, seasons and sensing regimes would strengthen understanding of how the framework generalizes under diverse plant-environment interactions. Finally, deeper analysis of abstention triggers, confidence thresholds and follow-up actions could support practical guidelines for when to defer recommendations, when to apply conservative defaults and when to initiate targeted strip trials or additional sensing to improve decision confidence.

## Data Availability

The datasets presented in this study can be found in online repositories. The names of the repository/repositories and accession number(s) can be found in the article/**Supplementary Material**.

## Supplementary material

Supplementary Figure 1 Smart sensing-enabled field operations relevant to plant-environment management and sustainable phytoprotection: tractor-mounted sensor-driven variable-rate fertilization and UAV-based precision spraying.

Supplementary Figure 2 Overall study flow linking smart sensing-relevant data, crop-response modeling, uncertainty calibration and risk-aware nitrogen decision support for sustainable plant-environment management.

Supplementary Table 1 Representative prior work in smart sensing-enabled nitrogen management, the remaining reliability gap and how the proposed framework addresses it.

Supplementary Figure 3 Literature-review taxonomy for smart sensing-enabled precision nitrogen management: (1) point-prescription streams, including point yield→EONR, crop/system models and remote sensing with onfarm trials; (2) uncertainty literature, including quantile and conformal methods; (3) the decision-reliability gap; and (4) the proposed framework that propagates conformal yield uncertainty to profit bounds and selects nitrogen rates through a lower-confidence-bound rule with abstention under low confidence.

Supplementary Table 2 Feature-wise missingness rates before preprocessing. Reporting these rates clarifies whether imputation is a minor data-cleaning step or a material modeling assumption for particular variables.

Supplementary Table 3 Data quality and split summary. All preprocessing statistics and encoders are fit on the training split only, then applied to calibration and test splits to avoid leakage.

Supplementary Table 4 Distribution of group sizes across train, calibration and test partitions. Reporting these statistics clarifies whether the region-crop-season grouping is balanced or dominated by a small number of large groups.

Supplementary Figure 4 Schematic of split conformal prediction for yield uncertainty. A trained yield model produces point predictions, residuals on a held-out calibration set are used to compute a conformal quantile and this quantile symmetrically expands test-time predictions to form finite-sample valid yield intervals.

Supplementary Table 5 Baseline economic parameters used in profit modeling. These values define the reference operating condition for the main experiments and are perturbed in the sensitivity analysis.

Supplementary Figure 5 Conceptual illustration of the risk-aware fertilizer prescription rule. For a given context *x*, multiple candidate nitrogen rates are evaluated through profit intervals induced by predictive uncertainty. The selected prescription maximizes the lower confidence bound of context-dependent profit, while pointestimate-optimal rates are visually contrasted. When uncertainty is excessive, the framework abstains from issuing a recommendation.

Supplementary Figure 6 Overview of the proposed risk-aware nitrogen prescription framework. Raw agronomic data undergo preprocessing and leakage-free group splitting into training, calibration and test sets. A nonlinear yield response model f(x,N) produces point predictions, which are augmented with conformal prediction to obtain calibrated yield intervals. These intervals are propagated through the profit model to form profit bounds, enabling lower-confidence-bound optimization and selective abstention under high uncertainty.

Supplementary Table 6 Baselines and comparators used in experiments. All methods share identical splits, nitrogen grids and economic parameters for fair comparison.

Supplementary Table 7 Ablation configurations used to quantify the contribution of individual components. Each ablation modifies only the listed element while keeping splits, models and evaluation protocol fixed.

Supplementary Table 8 Economic scenarios used in the sensitivity analysis.

Supplementary Table 9 Descriptive statistics after preprocessing, summarizing variability in yield, nitrogen and covariates used in modeling and decision-making.

Supplementary Figure 7 Correlation heatmap of numerical features used in the yield model.

Supplementary Figure 8 Yield versus nitrogen by region, illustrating heterogeneity in response patterns across regions.

Supplementary Table 10 Leakage-free split summary under group-based partitioning used for training, calibration and evaluation.

Supplementary Figure 9 Distributional consistency under group-based splitting. (a) Empirical nitrogen-rate distributions across training, calibration and test splits. (b) Corresponding yield distributions, illustrating alignment across partitions and supporting robust uncertainty calibration.

Supplementary Table 11 Yield model comparison on the calibration split used to select the base predictor for conformal calibration and downstream prescriptions.

Supplementary Table 12 Conformal diagnostics on the test split, reporting nominal targets, empirical coverage and average interval width.

Supplementary Figure 10 Nominal versus achieved conformal coverage on the test split.

Supplementary Figure 11 Conditional coverage by groups under the leakage-free split at the 0.95 target.

Supplementary Table 13 Worst-group reliability summary across groupings, reporting mean and worst-group coverage and interval width at each target level.

Supplementary Figure 12 Conformal interval width versus difficulty drivers. Scatter plots show pointwise variation; binned plots summarize mean width across difficulty quantiles.

Supplementary Table 14 Uncertainty interval quality and corresponding decision performance on the test split.

Supplementary Table 15 Decision performance on the test split across prescription strategies.

Supplementary Figure 13 Profit versus coverage under selective recommendation, illustrating the reliability-coverage trade-off.

Supplementary Table 16 Selective recommendation behavior under different conformal target coverages. Higher target coverage increases the conformal quantile *q*_1−_*_α_*, which widens intervals and increases abstention.

Supplementary Figure 14 Abstention-rate heatmap across NDVI and rainfall bins on the test split.

Supplementary Table 17 Decision robustness by group on the test split, reporting subgroup sample size, aggregate outcomes, variability, paired per-sample differences relative to the expected-profit baseline and abstention across regions and crop types. Paired differences are computed on the same test samples within each group.

Supplementary Table 18 Ablation study and sensitivity to conformal target coverage, reporting how design choices affect profitability, prescription dispersion, abstention and distributional spread. Additional IQR and upper-tail summaries are included to distinguish broad-based variability increases from tail-driven effects.

Supplementary Table 19 Comparison with representative prior work on site-specific nitrogen decisions and uncertainty handling.
