# Supplementary figures and images for: Smart sensing-enabled risk-aware nitrogen prescriptions via conformal profit bounds for precision agriculture
Source: Front Plant Sci. 2026 Apr 27;17:1821003. doi: 10.3389/fpls.2026.1821003 (PMC13158193; doi:10.3389/fpls.2026.1821003)

**S1a**


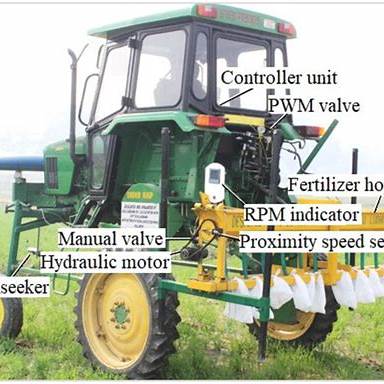


**S1b**


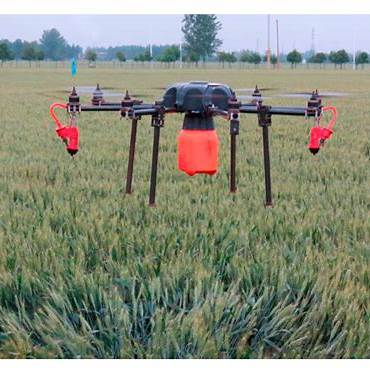


**S2**


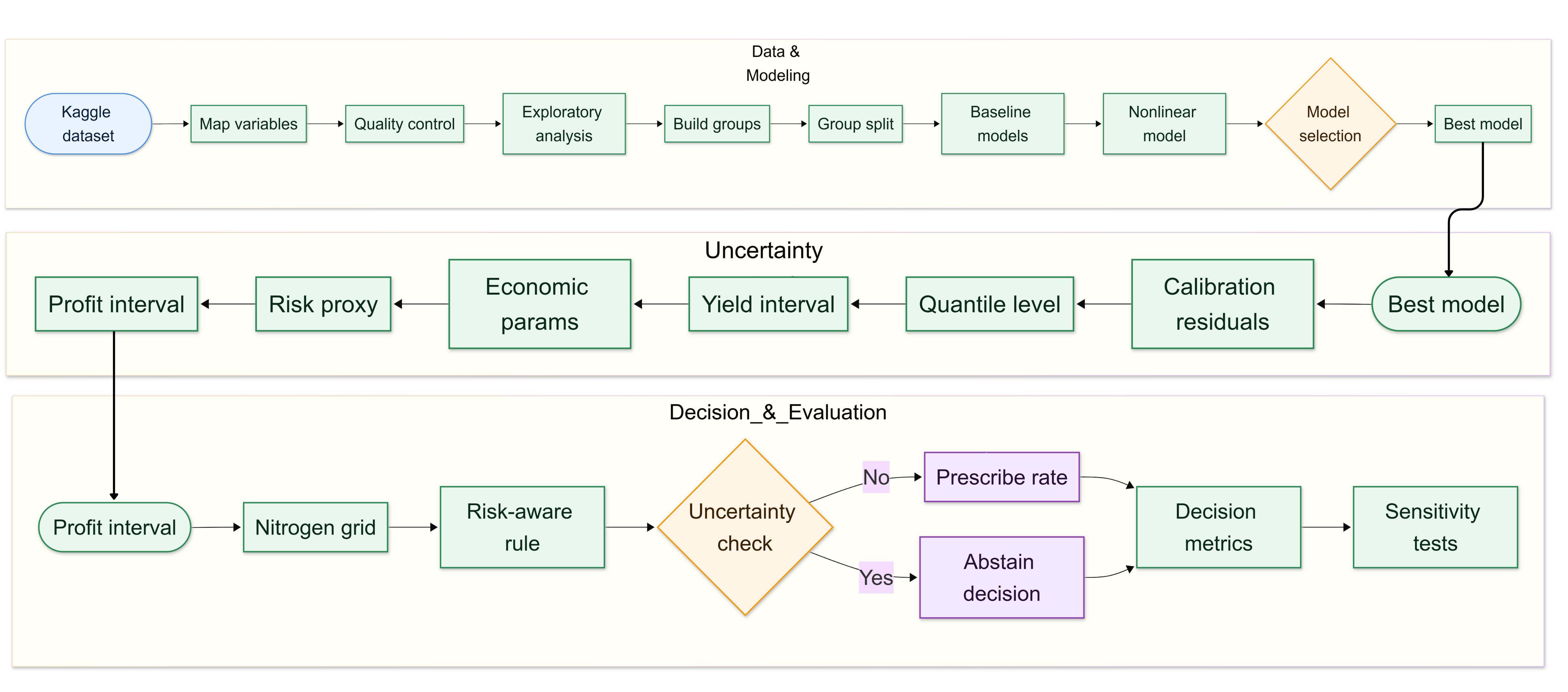


**S3**


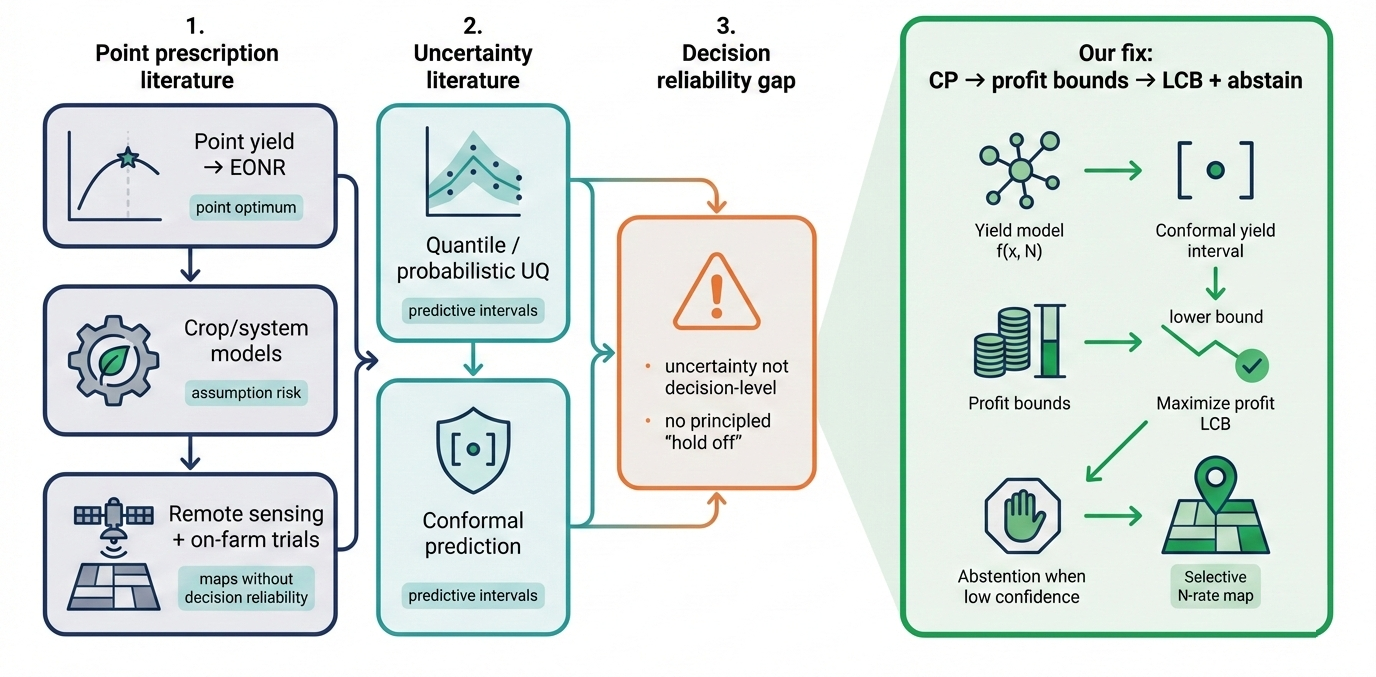


**S4**


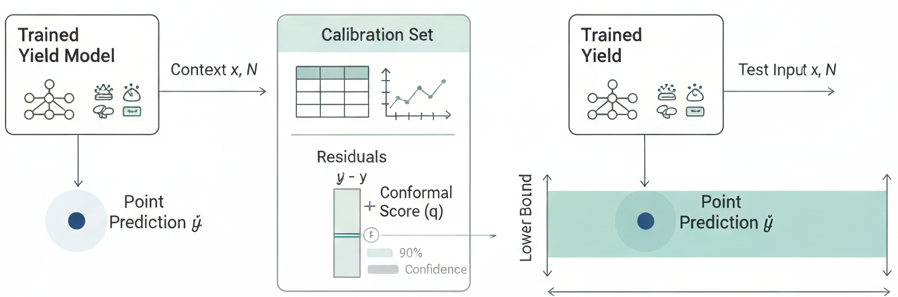


**S5**


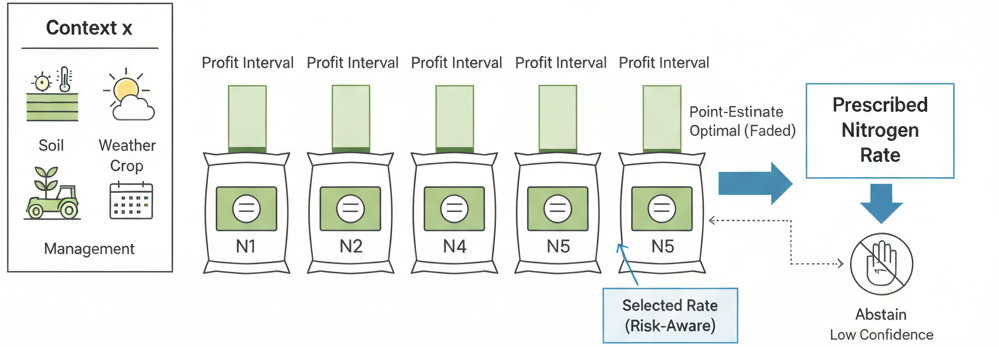


**S6**


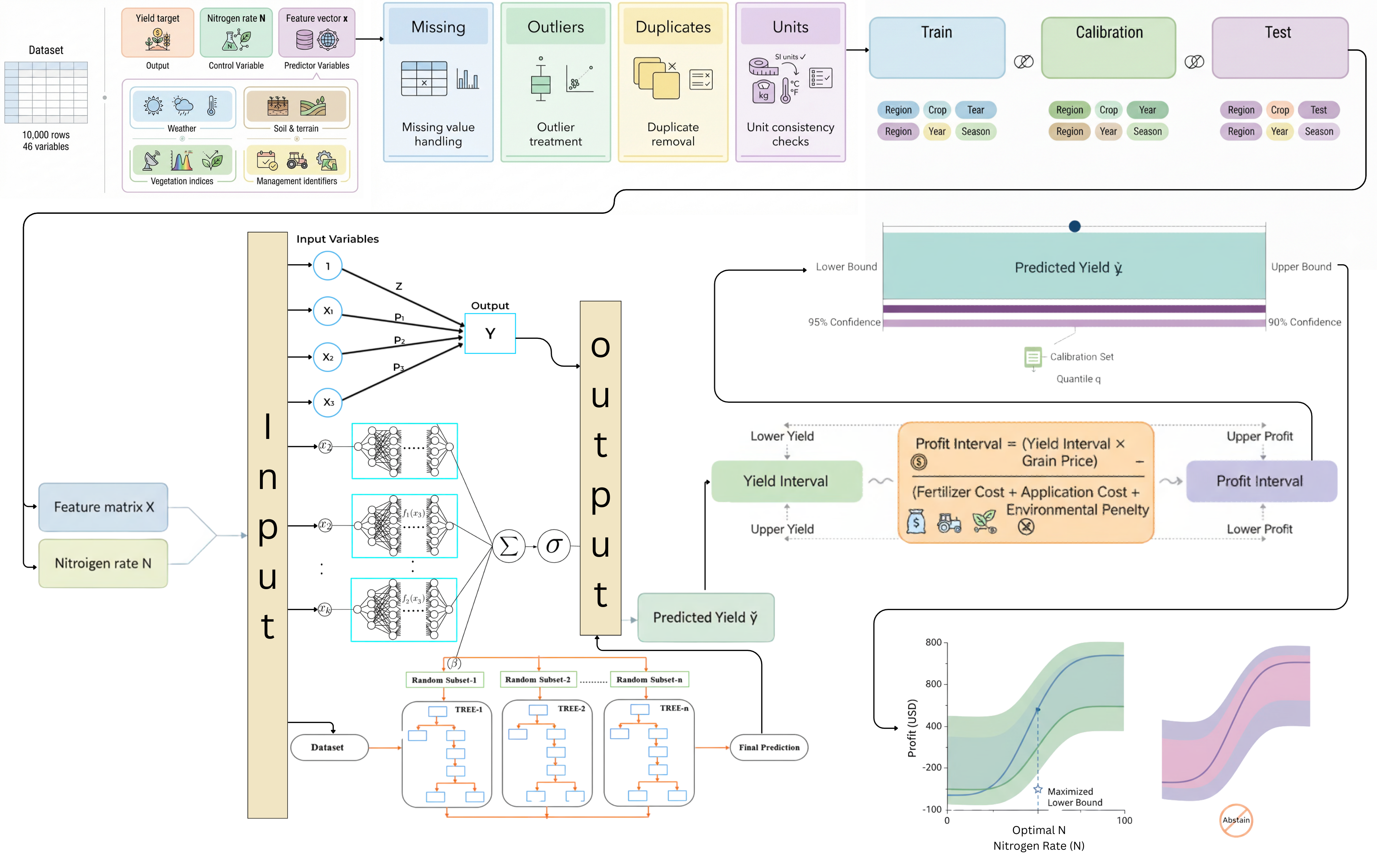


**S7**


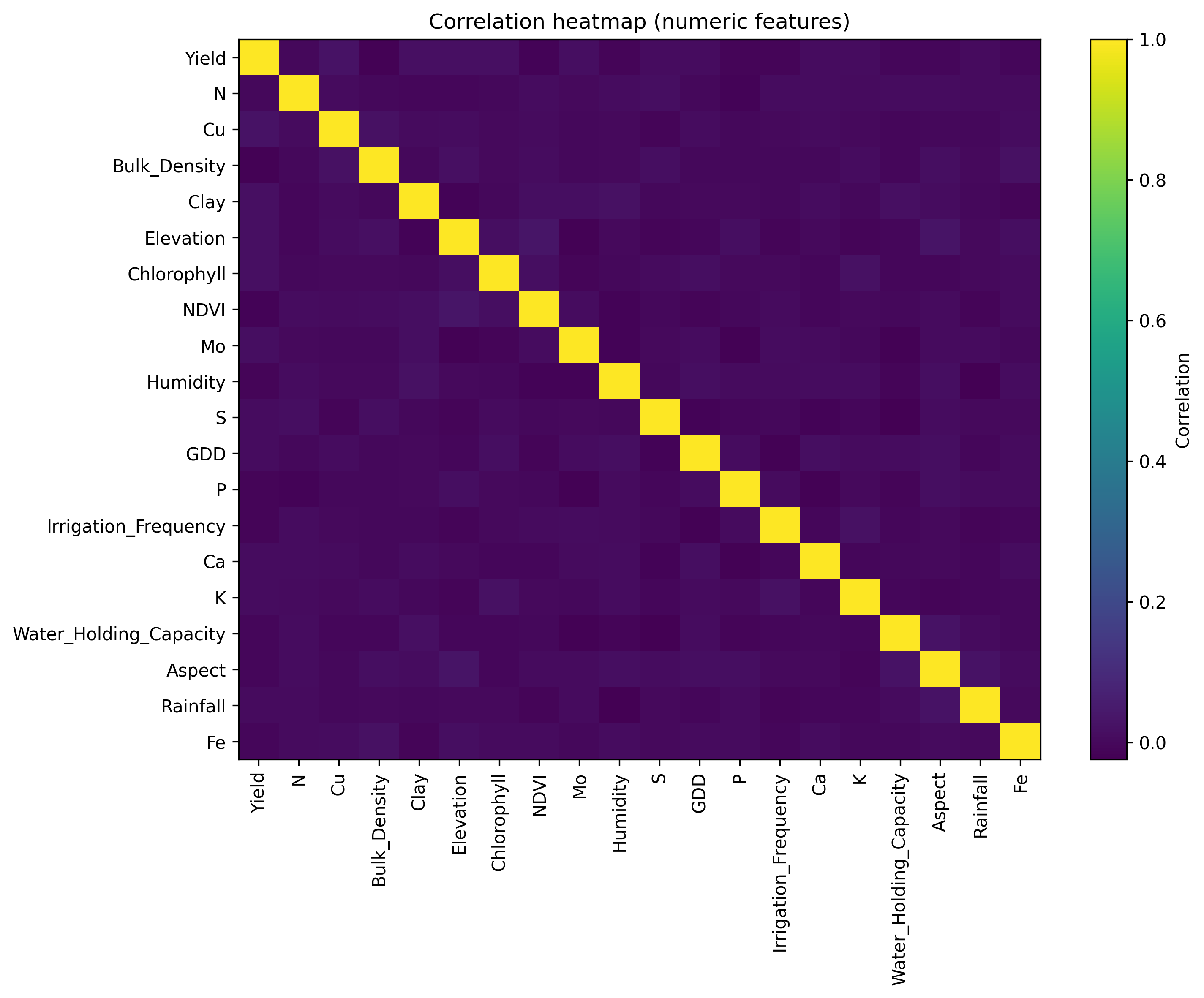


**S8**


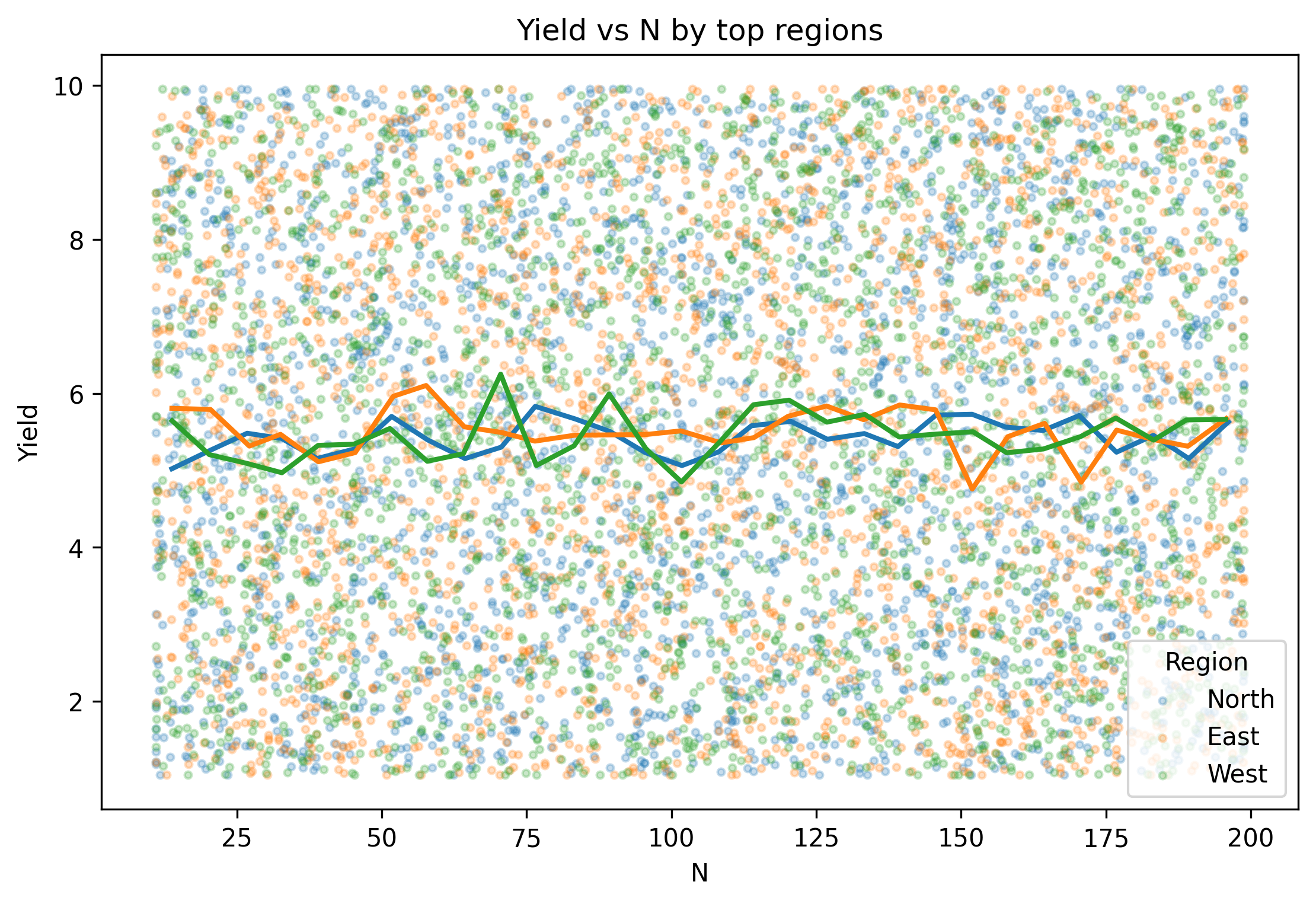


**S9a**


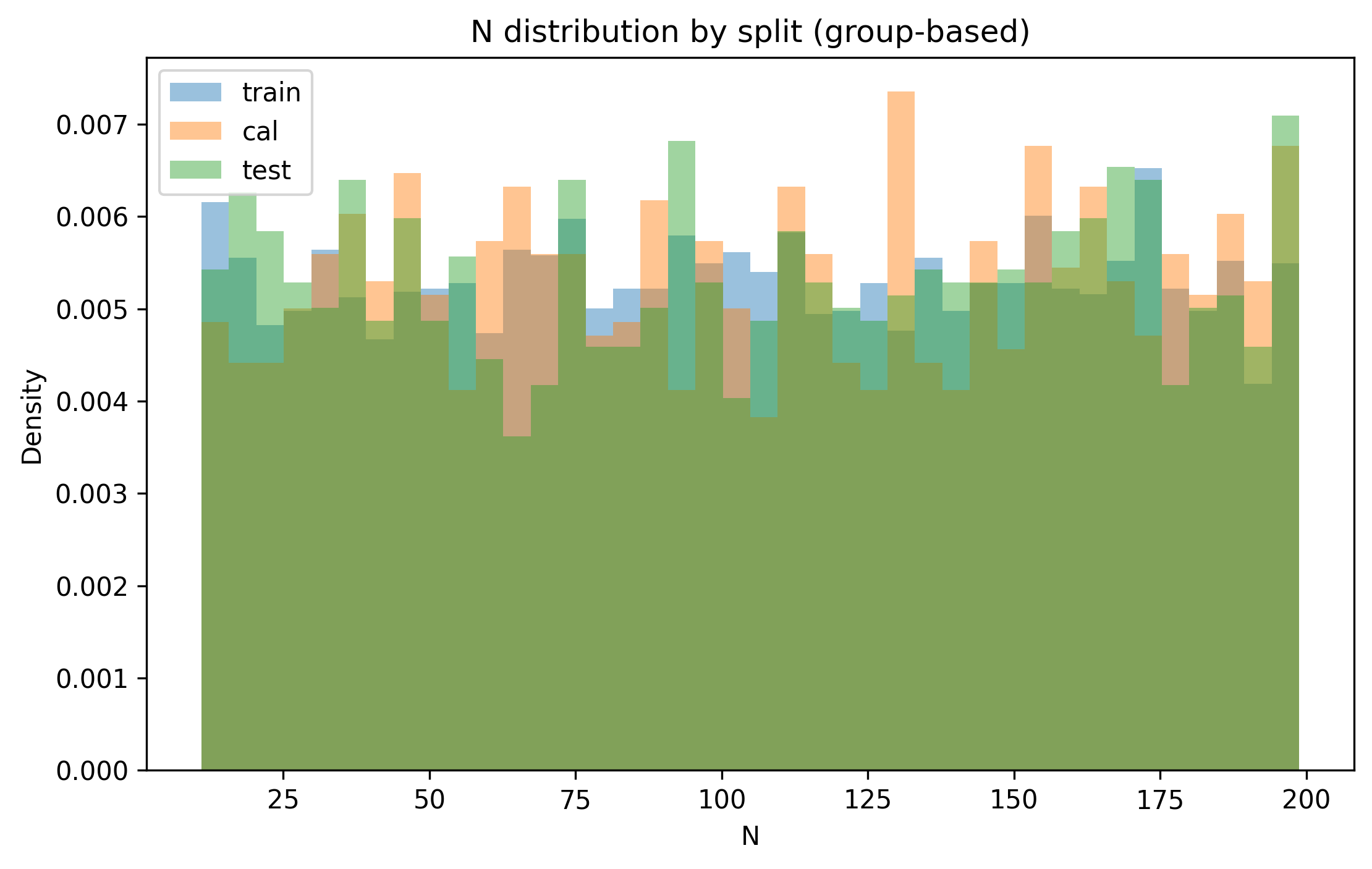


**S9b**


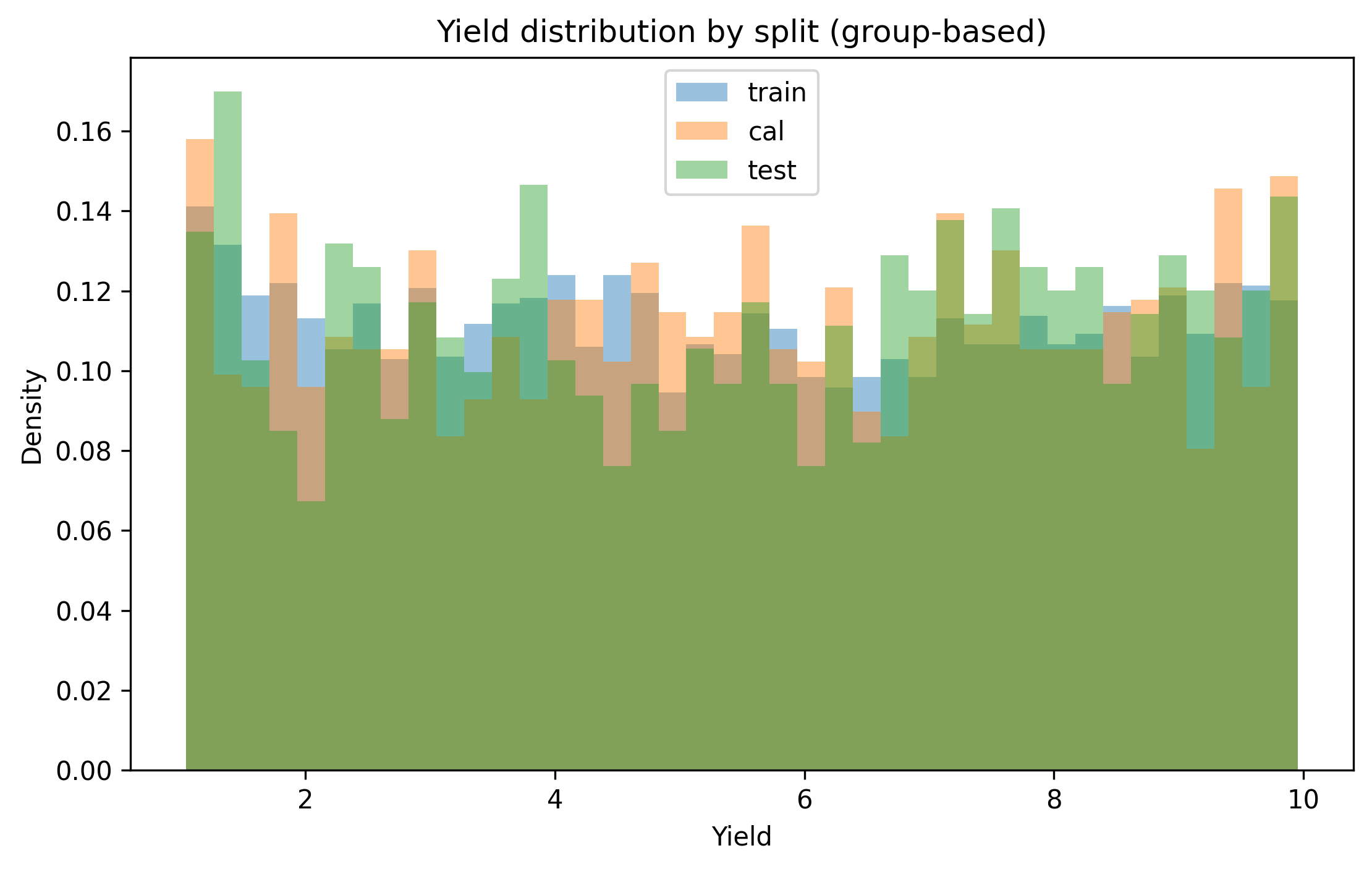


**S10**


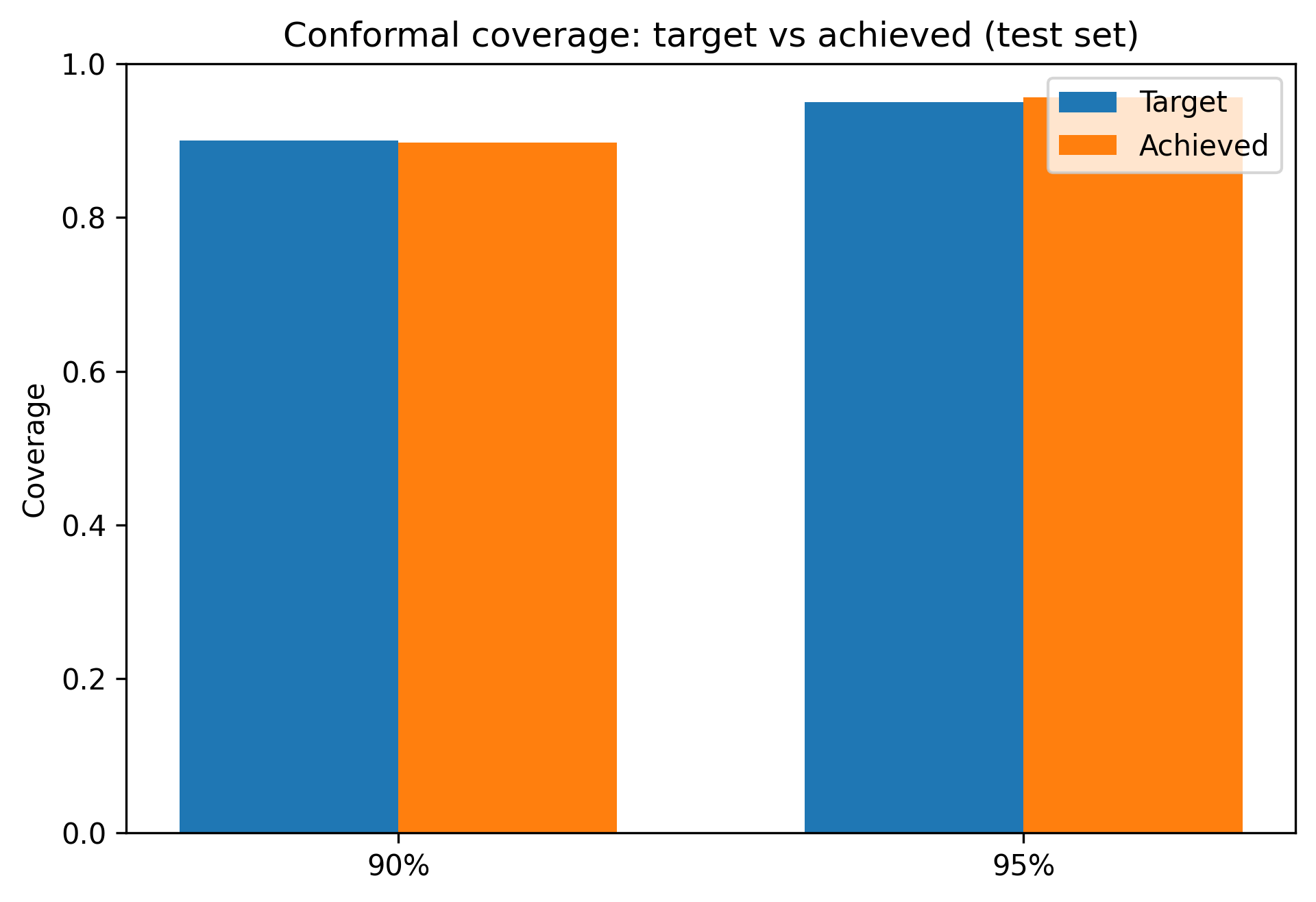


**S11a**


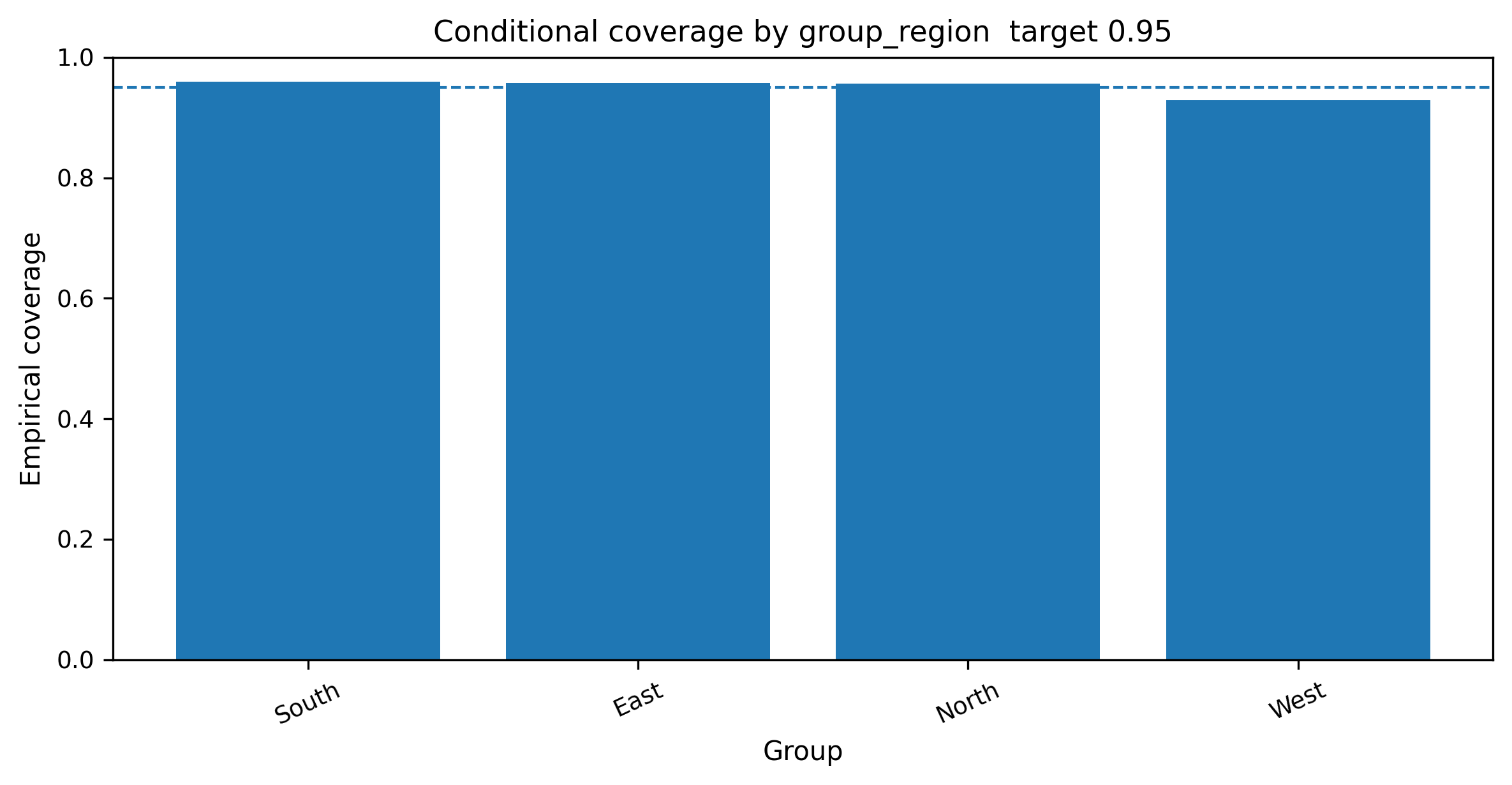


**S11b**


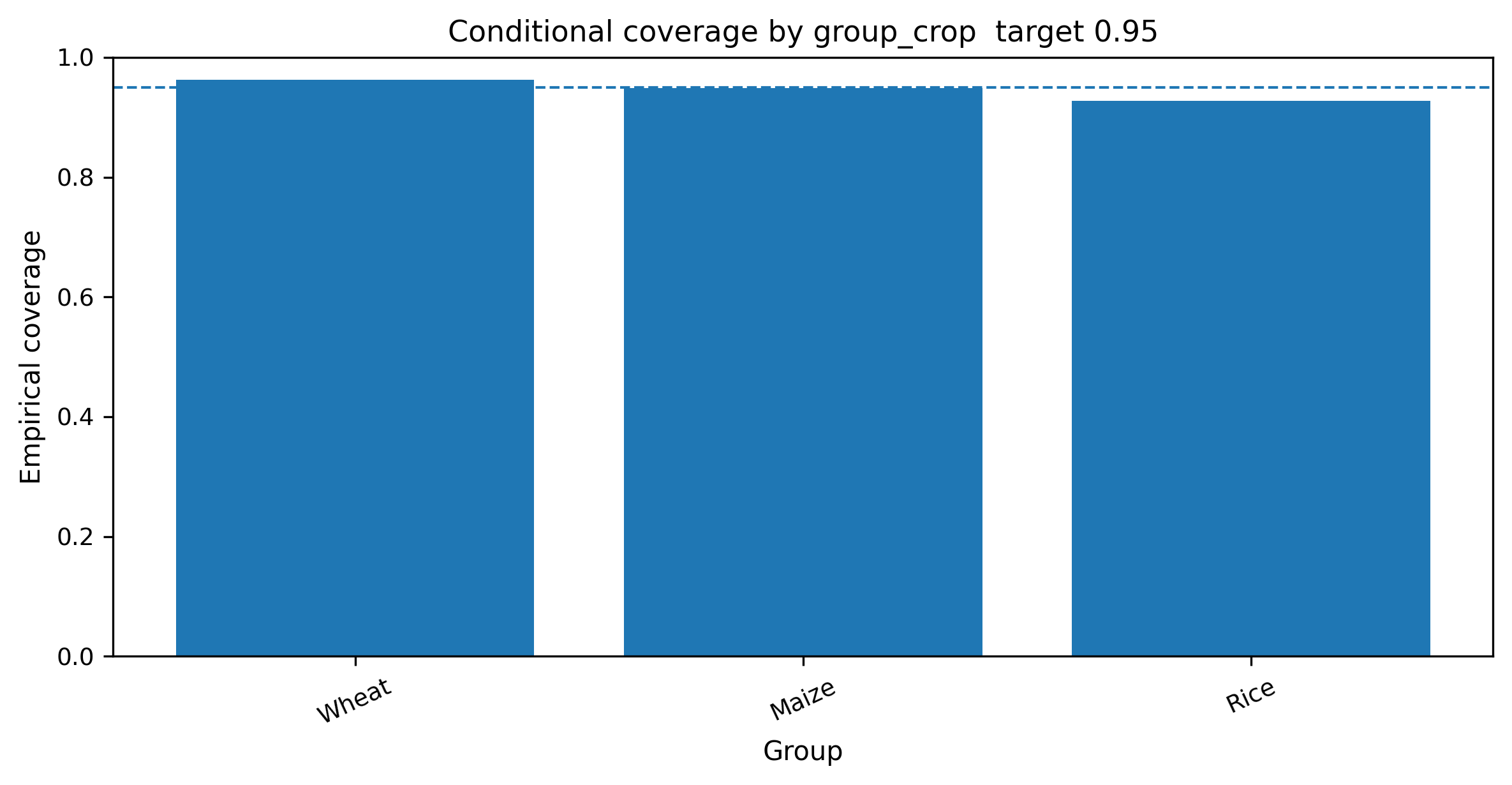


**S11c**


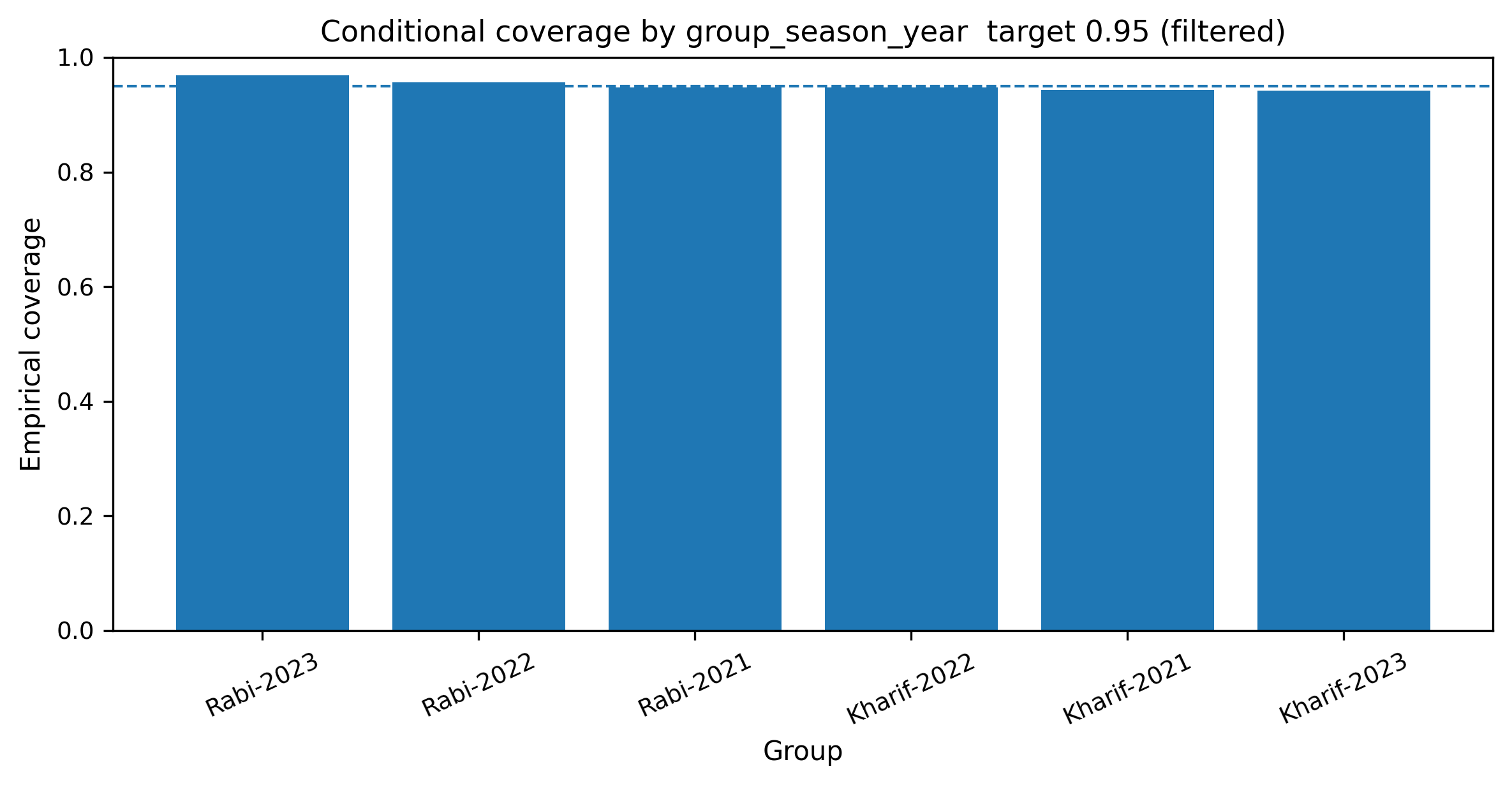


**S12a**


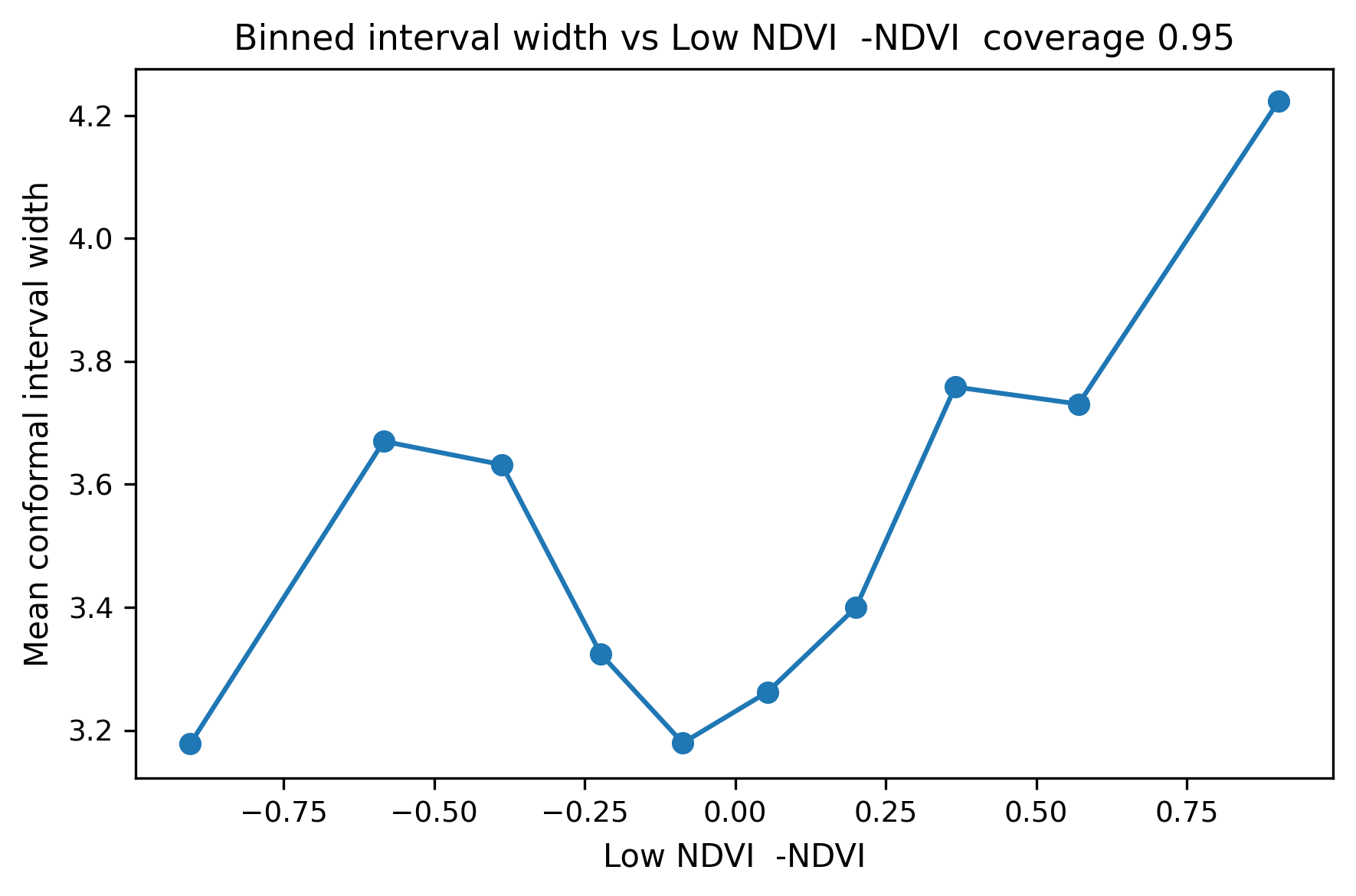


**S12b**


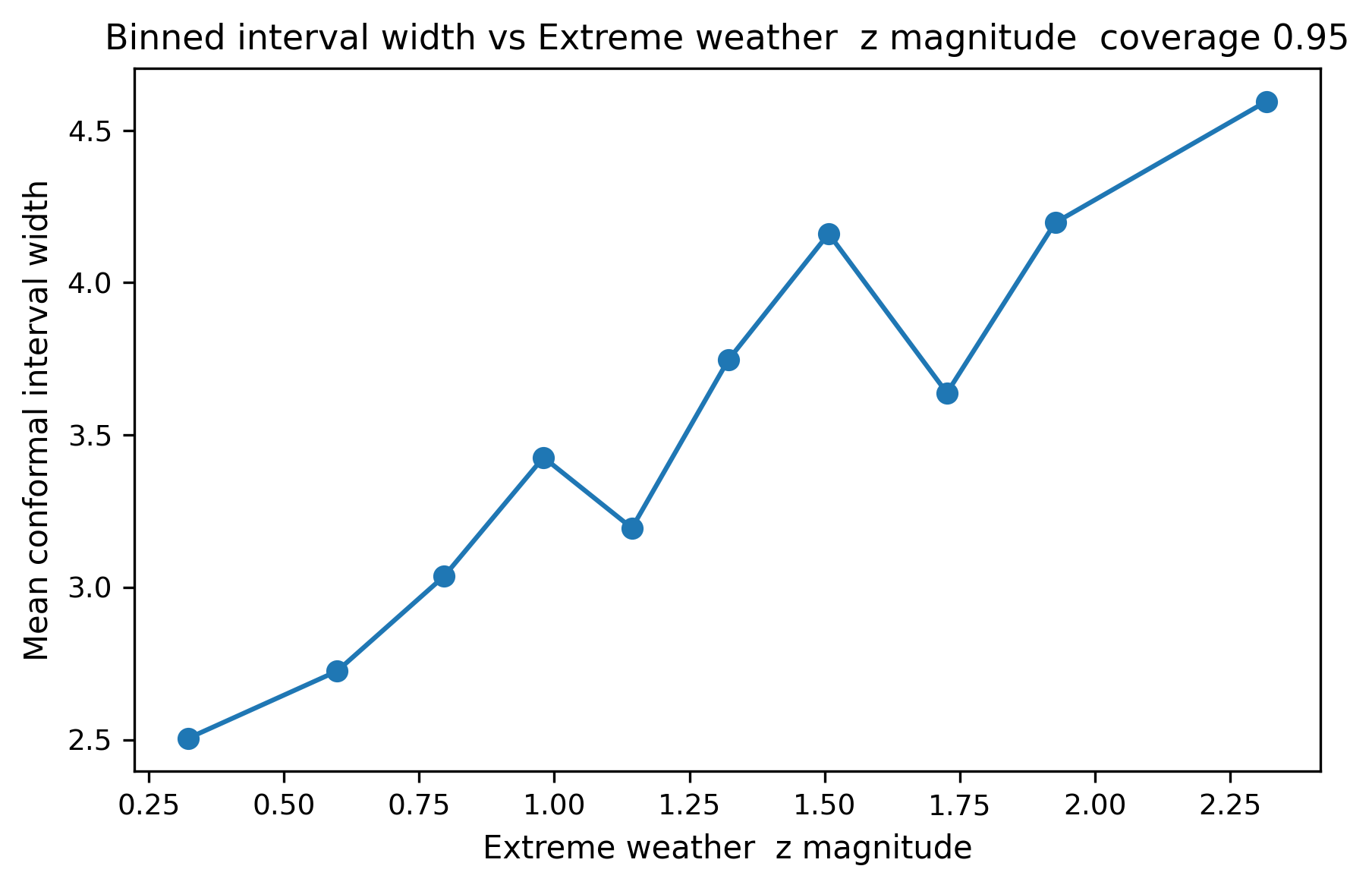


**S12c**


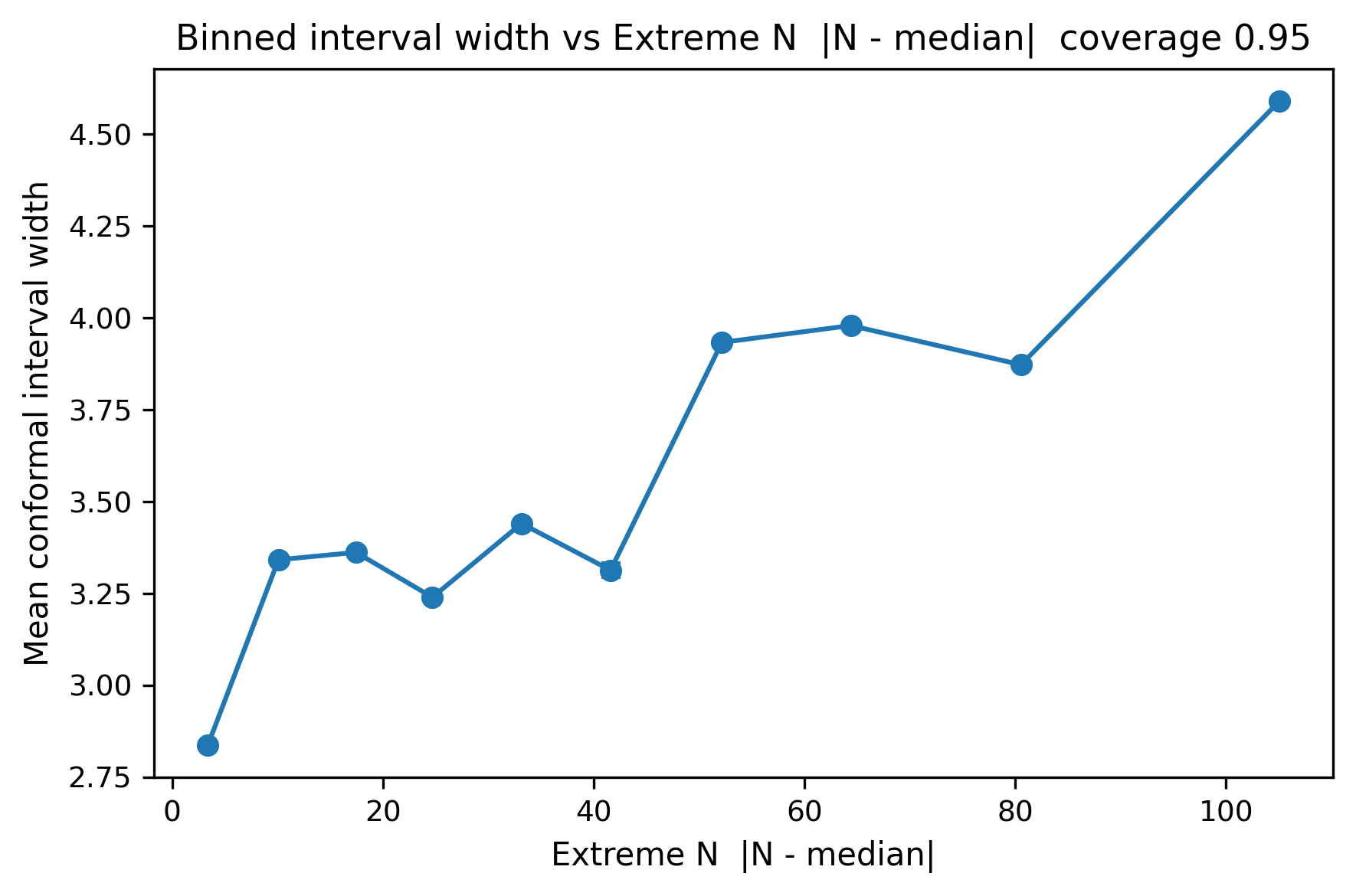


**S12d**


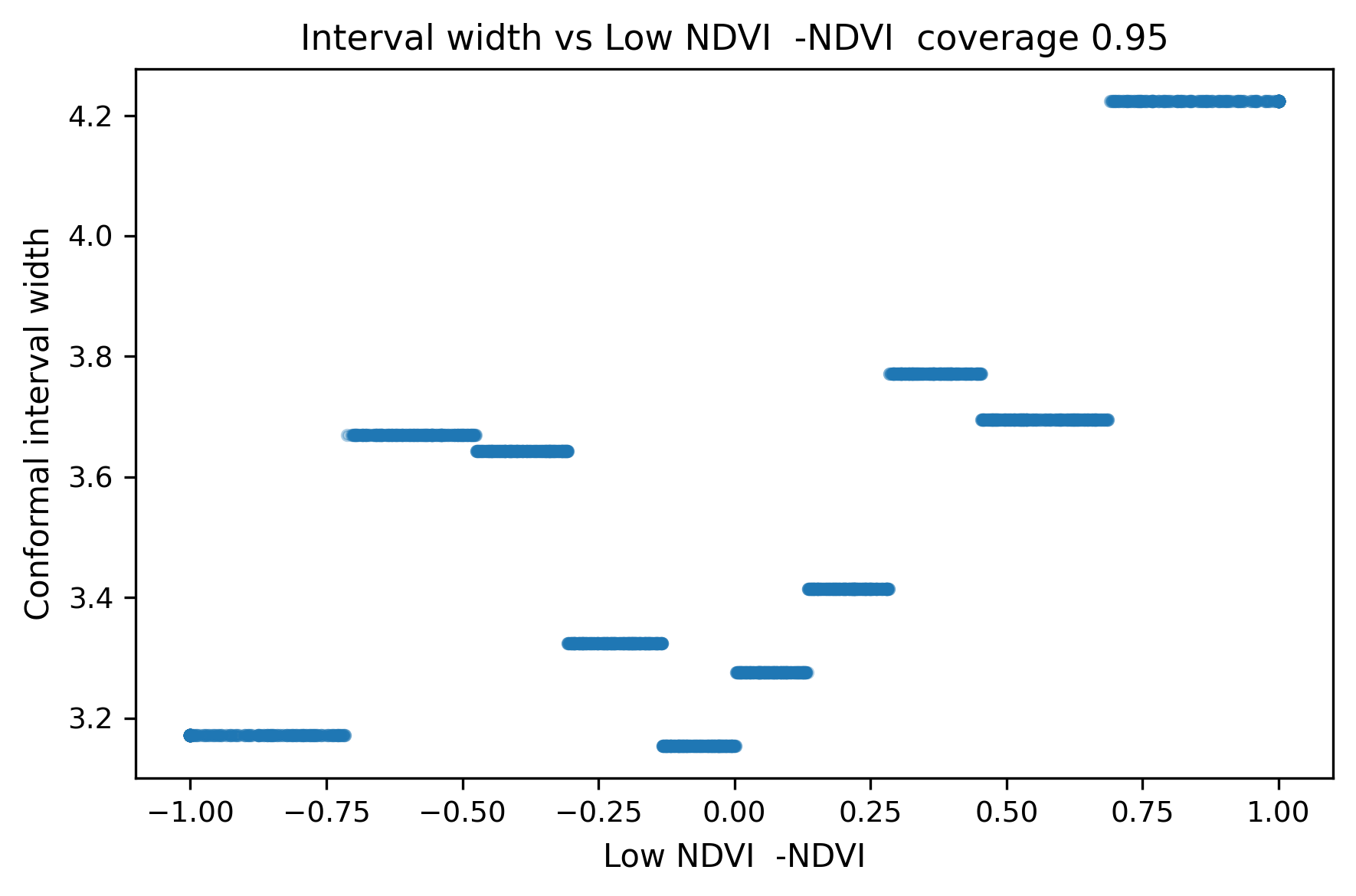


**S12e**


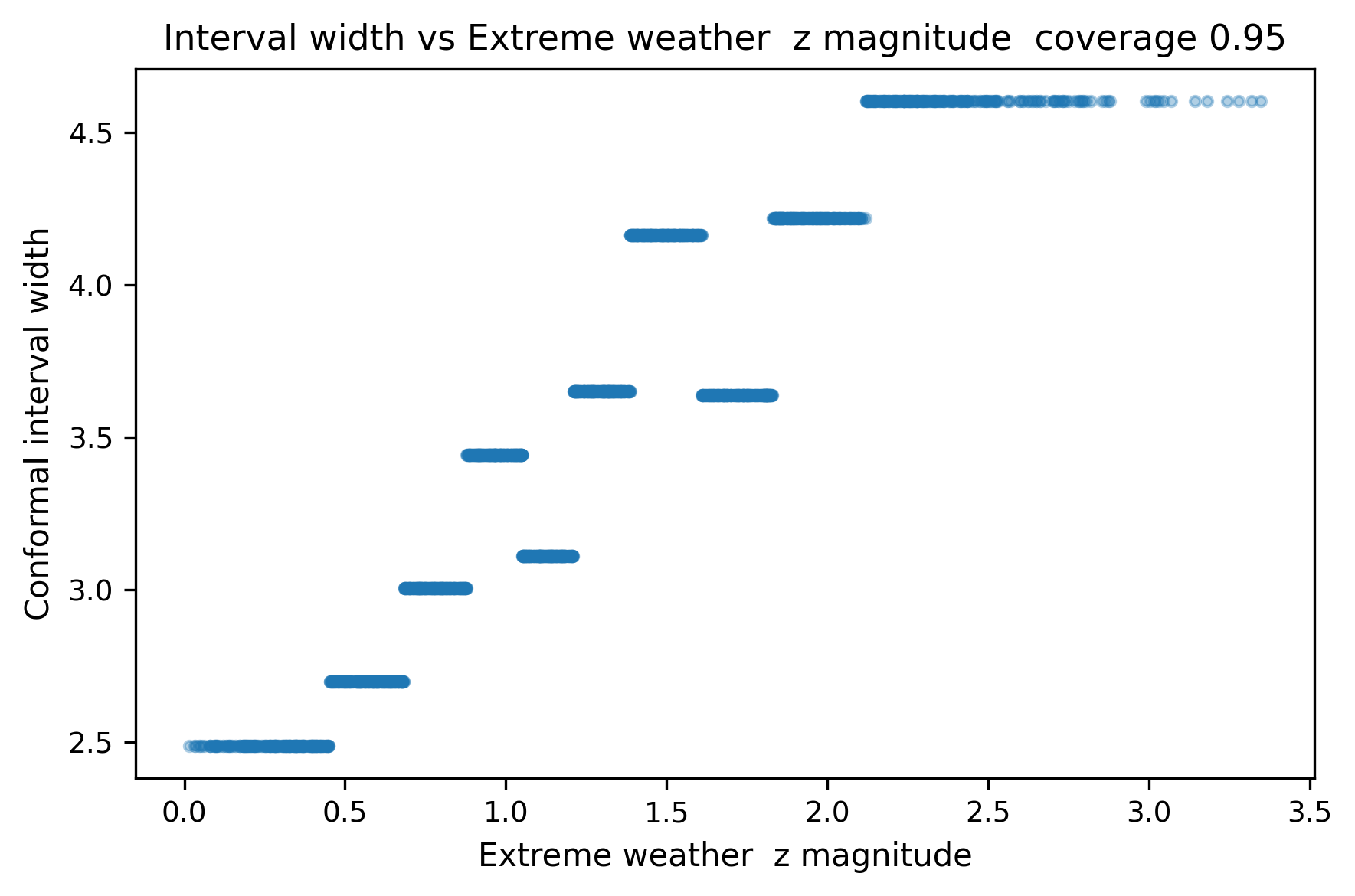


**S12f**


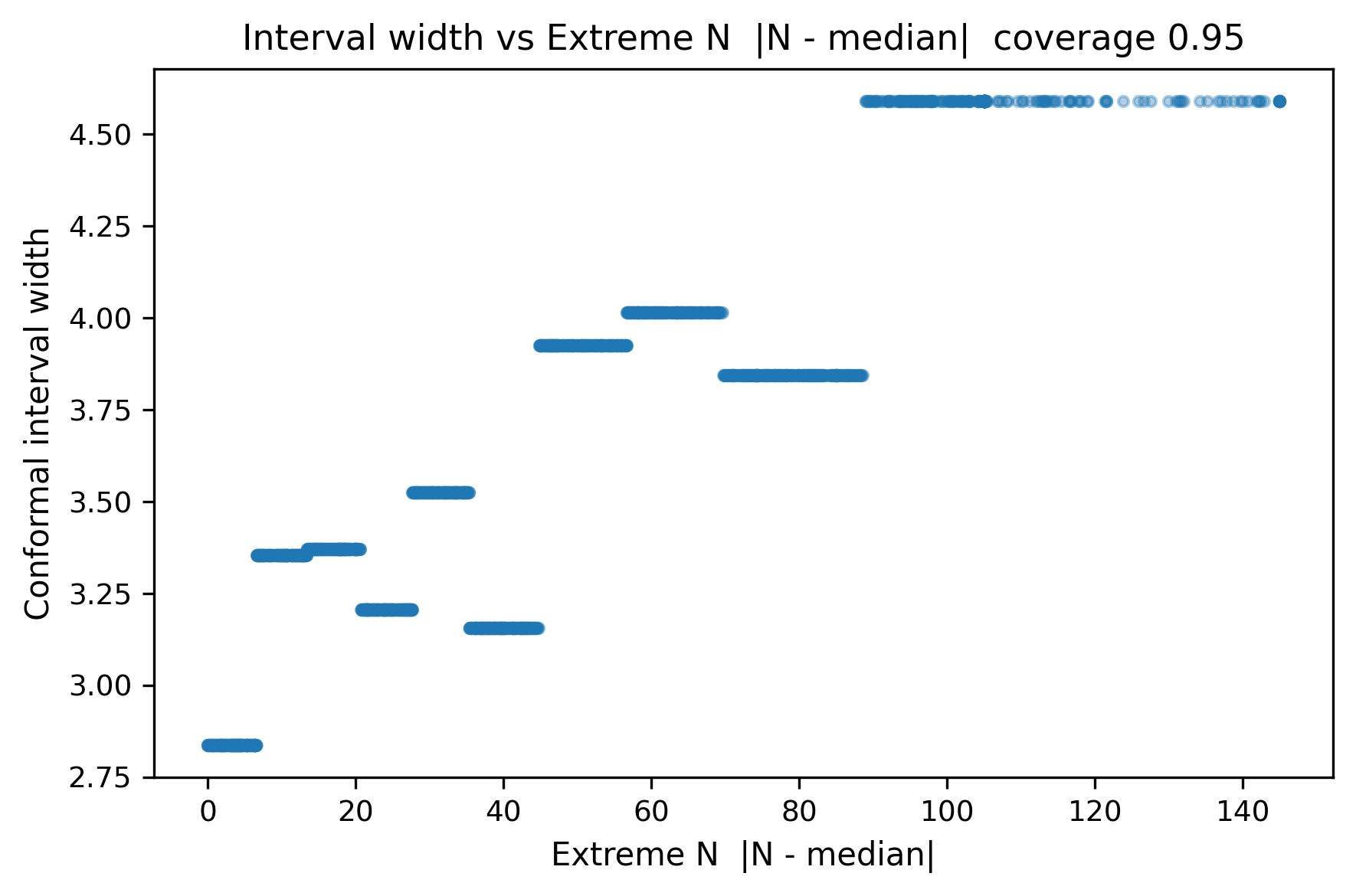


**S13**


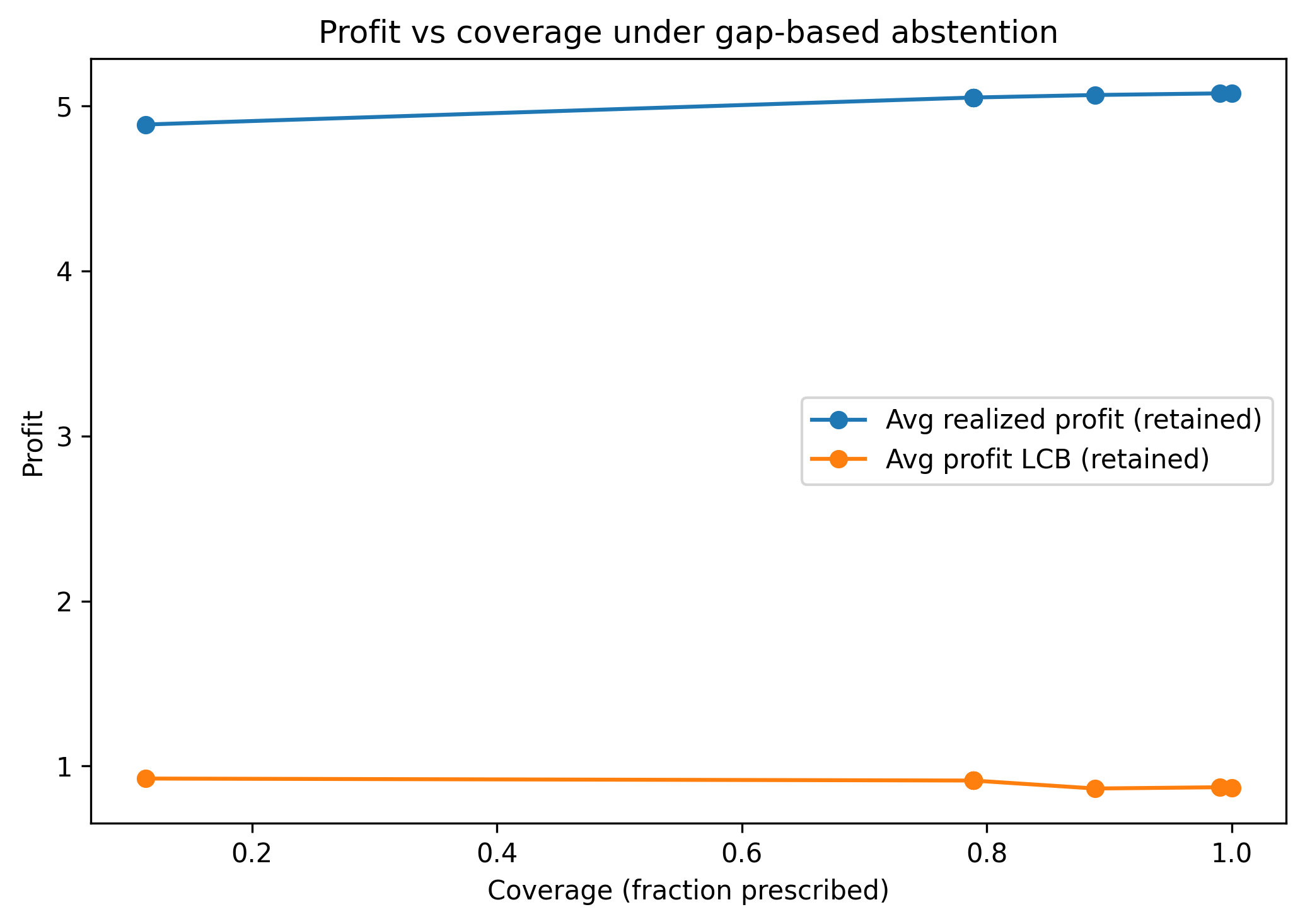


**S14**


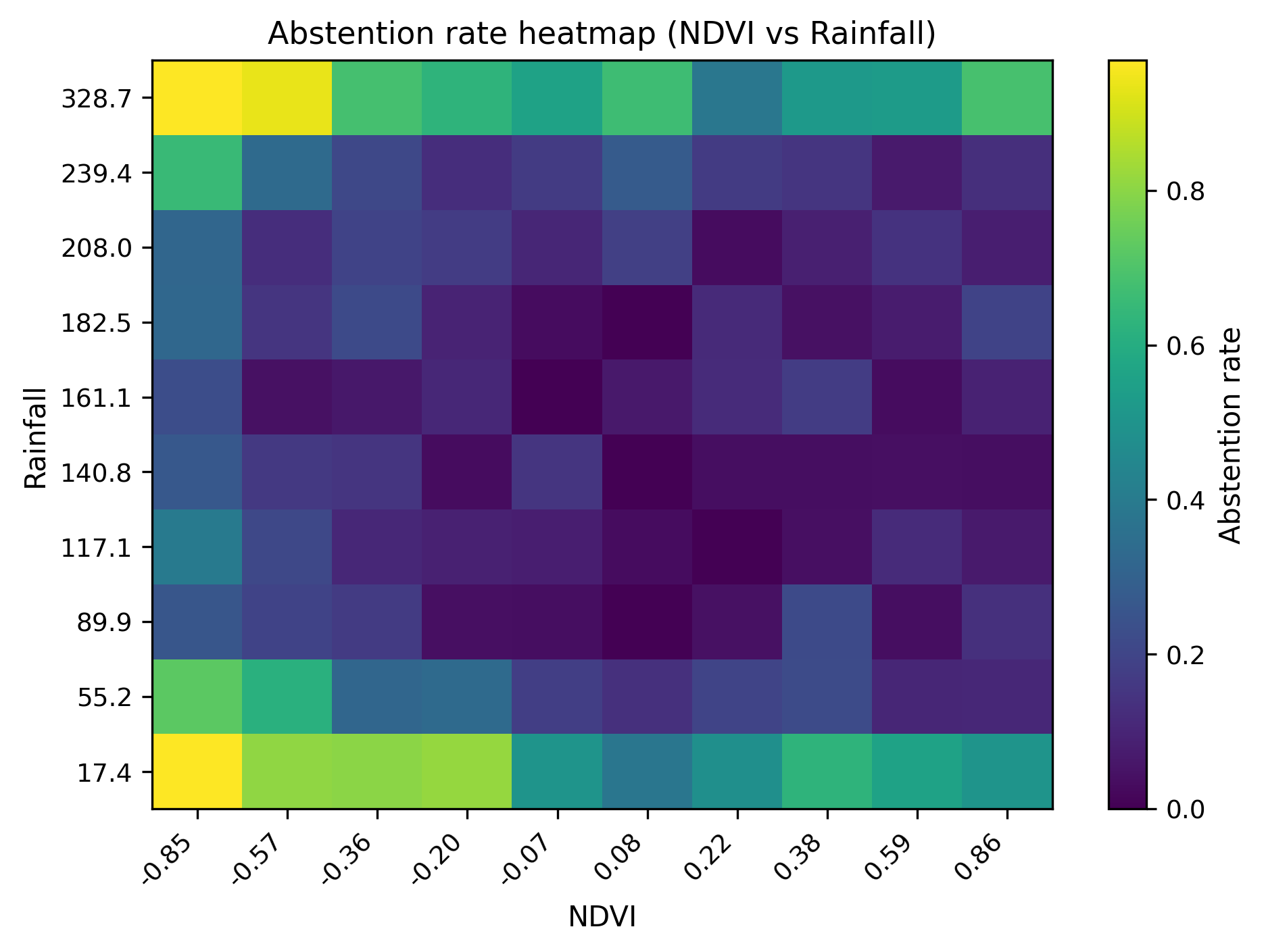

Supplement: Supplementary Figure 1 — Smart sensing-enabled field operations relevant to plant-environment management and sustainable phytoprotection: tractor-mounted sensor-driven variable-rate fertilization and UAV-based precision spraying. [file SupplementaryFile1.docx]
