# Supplementary material for: Smart sensing-enabled risk-aware nitrogen prescriptions via conformal profit bounds for precision agriculture
Source: Front Plant Sci. 2026 Apr 27;17:1821003. doi: 10.3389/fpls.2026.1821003 (PMC13158193; doi:10.3389/fpls.2026.1821003)
Supplement: Supplementary Table 1 — Representative prior work in smart sensing-enabled nitrogen management, the remaining reliability gap and how the proposed framework addresses it. [file Table1.docx]

| **Ref** | **Method** | **Gap** | **Our fix** |
| --- | --- | --- | --- |
| [28, 46, 18] | Point EONR from point yield models | Sensing-informed but no decision-level reliability and no downside control | Conformal prediction yield intervals, profit bounds, and profit lower confidence bound maximization |
| [29, 30] | Crop and systems models for optimum N | Model and assumption risk, with limited decision uncertainty quantification | Model-agnostic conformal prediction calibration and propagation to profit bounds |
| [47, 31, 33, 34] | Remote sensing and on-farm trials for prescription maps | Uncertainty not tied to prescriptions and weak support for cautious intervention under distribution shift | Group-split evaluation, lower confidence bound rule, and abstention under low confidence |
| [48, 39, 43, 49] | Quantile uncertainty quantification and conformal prediction in adjacent agricultural tasks | Mostly predictive reliability, with limited translation to decision-level control | Finite-sample conformal prediction and conversion of yield intervals to profit intervals |
| [37, 44] | Reject option and prescriptive machine learning | Rare application to nitrogen-rate selection and smart sensing field decisions | Abstention rule for indistinguishable or high-uncertainty rates |

# Table S1

# Table S2

| **Feature group** | **Feature** | **Missing (%)** | **Imputation rule** |
| --- | --- | --- | --- |
| Weather and climate | Temperature | 1.9 | Median from training split |
| Weather and climate | Rainfall | 2.7 | Median from training split |
| Soil and terrain | Soil pH | 3.4 | Median from training split |
| Soil and terrain | Organic carbon | 4.8 | Median from training split |
| Crop condition | NDVI | 5.9 | Median from training split |
| Crop condition | EVI | 6.3 | Median from training split |
| Management / categorical | Crop type | 0.4 | Unknown category from training split |
| Management / categorical | Irrigation frequency | 1.6 | Unknown category from training split |

# Table S3

| **Component** | **Procedure** | **Artifact** |
| --- | --- | --- |
| Missing values | Feature-wise missingness quantified before preprocessing; median imputation for numeric features and unknown category for categorical variables | Missingness summary table and imputer fit on training split |
| Outliers | Quantile-based winsorization for numeric predictors and yield | Clip bounds estimated from training split |
| Encoding | One-hot encoding for categorical variables | Encoder fit on training split |
| Scaling | Standardization using Eq. S3 with ϵ = 10−8 | $(\mu tr, \sigma tr, \epsilon)$ from training split and fixed preprocessing specification |
| Duplicates | Exact match removal after type normalization | Cleaned index list |
| Split strategy | Deterministic group split using canonical key in Eq. S4, SHA-256 hash in Eq. S5, lexicographic sorting of unique groups, fixed seed 42, target proportions 60/20/20, and summary of group-size distribution in Table S4; pooled marginals used only as a sanity check, not as the main justification of split validity | Train, calibration, and test group sets with reproducible group IDs and group-size statistics |

# Table S4

| **Split** | **# Groups** | **Min** | **Median** | **Mean** | **IQR** | **Max** | **# Groups < 8** |
| --- | --- | --- | --- | --- | --- | --- | --- |
| Train | 420 | 5 | 14 | 14.29 | 5 | 30 | 14 |
| Calibration | 140 | 6 | 14 | 14.29 | 5 | 28 | 4 |
| Test | 140 | 5 | 14 | 14.29 | 6 | 29 | 5 |

# Table S5

| **Symbol** | **Meaning** | **Unit** | **Baseline value** |
| --- | --- | --- | --- |
| $\left( p_{g} \right)$ | Grain price per unit yield | Currency per yield unit | 1.00 |
| $\left( c_{N} \right)$ | Nitrogen cost per unit application rate | Currency per rate unit | 0.50 |
| $\left( c_{\text{app}} \right)$ | Fixed application cost per decision | Currency | 10.0 |
| $\left( \lambda\right)$ | Environmental penalty weight applied to surplus proxy | Currency per proxy unit | 0.20 |
| $\left( \tilde{N}\left( x \right) \right)$ | Context-dependent reference nitrogen rate used in Eq. S14 | Rate unit | Training-split crop-region mean nitrogen; fallback = 105.19 |

# Table S6

| **Method** | **Key idea** | **Output type** |
| --- | --- | --- |
| Uniform fertilizer | Constant nitrogen rate equal to the training-set mean | Single rate |
| Expected profit | Maximize point profit using the same base yield predictor as the main pipeline and the economic rule in Eq. S21 | Site-specific point rate |
| Point estimate EONR | Grid-search profit optimum without uncertainty bounds using the same base yield predictor and Eq. S13 | Site-specific point rate |
| Quantile regression intervals | Learn lower and upper conditional quantiles for yield using $\left( \left( \alpha/2, 1-\alpha/2 \right) \right)$ | Yield and profit intervals |
| Ensemble uncertainty | (M = 10) independently fitted tree-ensemble models with bootstrap resampling; interval from empirical prediction distribution | Yield and profit intervals |
| Dropout uncertainty | Monte Carlo dropout using a multilayer perceptron regressor, rate (p = 0.20) and (T = 50) stochastic forward passes | Yield and profit intervals |

# Table S7

| **Ablation** | **Modification** |
| --- | --- |
| No environmental penalty | Set ($\lambda$= 0) in Eq. S13 |
| No abstention | Always prescribe using Eq. S20; disable selective rule |
| Weather only | Use weather and climate covariates only |
| Soil and terrain only | Use soil chemistry and terrain covariates only |
| Crop condition only | Use crop-condition indices only |
| Full features | Use all feature groups including indices and management |
| Coverage level | Compare (1 - $\alpha$ = 0.90) versus (1 - $\alpha$ = 0.95) in Eq. S12 |

# Table S8

| **Scenario** | $\left( \boldsymbol{p}_{\boldsymbol{g}} \right)$ | $\left( \boldsymbol{c}_{\boldsymbol{N}} \right)$ | $\left( \boldsymbol{c}_{\text{app}} \right)$ | $\left( \boldsymbol{\lambda} \right)$ | **Expected effect on** $\left( \boldsymbol{N}^{\boldsymbol{*}} \right)$ |
| --- | --- | --- | --- | --- | --- |
| Baseline | 1.00 | 0.50 | 10.0 | 0.20 | Reference operating condition used in the main experiments |
| Low grain price | 0.80 | 0.50 | 10.0 | 0.20 | Reduced marginal value of yield is expected to shift prescriptions toward smaller $\left( N^{*} \right)$ |
| High grain price | 1.20 | 0.50 | 10.0 | 0.20 | Higher crop value is expected to justify larger $\left( N^{*} \right)$ when yield response remains favorable |
| High nitrogen cost | 1.00 | 0.60 | 10.0 | 0.20 | Higher marginal input cost penalizes application and is expected to reduce $\left( N^{*} \right)$ |
| High application cost | 1.00 | 0.50 | 15.0 | 0.20 | Larger fixed cost reduces the attractiveness of aggressive recommendations, especially where expected gains are small |
| High environmental weight | 1.00 | 0.50 | 10.0 | 0.40 | Stronger surplus penalty in Eq. S14 is expected to shift prescriptions toward smaller $\left( N^{*} \right)$ |
| Combined adverse economics | 0.80 | 0.60 | 15.0 | 0.40 | Simultaneously lower crop value and higher economic or environmental penalties should produce the most conservative prescriptions |

# Table S9

| **Variable** | **Mean** | **Std. Dev.** | **P25** | **Median** | **P75** |
| --- | --- | --- | --- | --- | --- |
| Yield | 5.470 | 2.633 | 3.182 | 5.445 | 7.779 |
| N | 105.189 | 54.375 | 58.196 | 105.025 | 152.900 |
| Temperature | 24.825 | 8.627 | 17.390 | 24.776 | 32.200 |
| Rainfall | 150.015 | 86.016 | 76.124 | 150.620 | 223.402 |
| Humidity | 60.272 | 17.355 | 45.237 | 60.354 | 75.389 |
| pH | 6.491 | 1.158 | 5.487 | 6.476 | 7.499 |
| EC | 1.299 | 0.693 | 0.697 | 1.307 | 1.900 |
| OC | 1.052 | 0.548 | 0.585 | 1.051 | 1.531 |
| NDVI | -0.002 | 0.577 | -0.505 | -0.001 | 0.496 |
| EVI | -0.004 | 0.576 | -0.504 | -0.012 | 0.502 |
| LAI | 2.993 | 1.723 | 1.503 | 2.987 | 4.485 |
| Chlorophyll | 30.053 | 11.540 | 19.903 | 29.918 | 40.090 |

# Table S10

| **Split** | **Samples** | **Groups** | **Percentage (%)** |
| --- | --- | --- | --- |
| Training | 6000 | 420 | 60.0 |
| Calibration | 2000 | 140 | 20.0 |
| Test | 2000 | 140 | 20.0 |
| Total | 10000 | 700 | 100.0 |

# Table S11

| **Model** | **RMSE** | **MAE** |
| --- | --- | --- |
| Ridge Regression | 1.982 | 1.543 |
| Additive Baseline | 1.764 | 1.402 |
| Tree Ensemble | 1.531 | 1.214 |

# Table S12

| **Target Coverage** | **Empirical Coverage** | **Avg. Interval Width** |
| --- | --- | --- |
| 0.90 | 0.912 | 2.847 |
| 0.95 | 0.963 | 3.421 |

# Table S13

| **Grouping** | **Target** | **Mean cov.** | **Worst cov.** | **Mean width** | **Worst width** |
| --- | --- | --- | --- | --- | --- |
| Region | 0.90 | 0.903 | 0.882 | 2.847 | 3.210 |
| Region | 0.95 | 0.952 | 0.931 | 3.421 | 3.980 |
| Crop type | 0.90 | 0.899 | 0.876 | 2.847 | 3.150 |
| Crop type | 0.95 | 0.948 | 0.923 | 3.421 | 3.910 |
| Season-Year | 0.90 | 0.905 | 0.884 | 2.847 | 3.280 |
| Season-Year | 0.95 | 0.951 | 0.929 | 3.421 | 4.050 |

# Table s14a panel

| **Method** | **Target** | **Empirical** | **Avg. width** | **Median width** | **IQR width** | **Abs. cal. error** |
| --- | --- | --- | --- | --- | --- | --- |
| Split conformal prediction | 0.90 | 0.912 | 2.847 | 2.65 | 0.90 | 0.012 |
| Split conformal prediction | 0.95 | 0.963 | 3.421 | 3.20 | 1.05 | 0.013 |
| Quantile regression intervals | 0.90 | 0.885 | 2.55 | 2.35 | 0.85 | 0.015 |
| Quantile regression intervals | 0.95 | 0.932 | 3.05 | 2.80 | 1.00 | 0.018 |
| Quantile random forest intervals | 0.90 | 0.918 | 3.10 | 2.90 | 1.10 | 0.018 |
| Quantile random forest intervals | 0.95 | 0.972 | 3.85 | 3.55 | 1.35 | 0.022 |
| Ensemble predictive intervals | 0.90 | 0.905 | 3.00 | 2.85 | 1.00 | 0.005 |
| Ensemble predictive intervals | 0.95 | 0.948 | 3.60 | 3.35 | 1.20 | 0.002 |
| Dropout predictive intervals | 0.90 | 0.872 | 2.40 | 2.20 | 0.80 | 0.028 |
| Dropout predictive intervals | 0.95 | 0.919 | 2.95 | 2.70 | 0.95 | 0.031 |

# Table s14b panel

| **Decision rule** | **Coverage** | **Mean profit** | **Profit std.** | **Mean N** | **N std.** | **Abstain (%)** |
| --- | --- | --- | --- | --- | --- | --- |
| Expected profit point rule, Eq. S21 | - | 4.68 | 1.19 | 121.34 | 18.76 | 0.0 |
| Split conformal LCB rule, Eq. S20 | 0.95 | 4.54 | 0.96 | 112.07 | 12.41 | 27.9 |
| Quantile LCB rule, Eq. S20 | 0.95 | 4.57 | 1.05 | 116.50 | 15.50 | 19.5 |
| Ensemble LCB rule, Eq. S20 | 0.95 | 4.52 | 0.99 | 111.20 | 13.20 | 24.0 |
| Dropout LCB rule, Eq. S20 | 0.95 | 4.60 | 1.14 | 119.00 | 17.00 | 13.0 |

# Table s15

| **Method** | **Mean Profit** | **Profit Std.** | **Mean N** | **N Std.** |
| --- | --- | --- | --- | --- |
| Uniform Rate | 4.12 | 0.88 | 105.19 | 0.00 |
| Point EONR | 4.61 | 1.24 | 124.03 | 21.57 |
| Expected Profit | 4.68 | 1.19 | 121.34 | 18.76 |
| Risk-Aware LCB | 4.54 | 0.96 | 112.07 | 12.41 |

# Table s16

| **Target Coverage** | **Abstention Rate (%)** | **Prescribed Fraction (%)** |
| --- | --- | --- |
| 0.90 | 18.4 | 81.6 |
| 0.95 | 27.9 | 72.1 |

# Table S17a Panel

| **Group** | **n** | **Mean profit** | **Profit std.** | **Mean Δπ** | **Median Δπ** | **Win (%)** | **Mean N** | **Mean ΔN** | **Abstain (%)** |
| --- | --- | --- | --- | --- | --- | --- | --- | --- | --- |
| North | 506 | 4.58 | 0.93 | -0.10 | -0.06 | 46.3 | 111.40 | -8.70 | 26.0 |
| South | 492 | 4.49 | 0.98 | -0.15 | -0.10 | 41.8 | 112.90 | -9.40 | 29.5 |
| East | 508 | 4.55 | 0.95 | -0.12 | -0.08 | 44.1 | 111.85 | -9.10 | 27.2 |
| West | 494 | 4.46 | 1.01 | -0.18 | -0.13 | 38.6 | 113.60 | -10.60 | 31.0 |

# Table S17b Panel

| **Group** | **n** | **Mean profit** | **Profit std.** | **Mean Δπ** | **Median Δπ** | **Win (%)** | **Mean N** | **Mean ΔN** | **Abstain (%)** |
| --- | --- | --- | --- | --- | --- | --- | --- | --- | --- |
| Wheat | 671 | 4.57 | 0.94 | -0.11 | -0.07 | 45.2 | 111.90 | -8.90 | 27.0 |
| Maize | 663 | 4.52 | 0.97 | -0.14 | -0.09 | 42.7 | 112.40 | -9.60 | 28.5 |
| Rice | 666 | 4.44 | 1.02 | -0.18 | -0.12 | 38.9 | 113.30 | -10.30 | 31.5 |

# Table S18a Panel

| **Configuration** | **Mean Profit** | **Profit Std.** | **Profit IQR** | **Profit P95** | **Mean N** | **N Std.** | **N IQR** | **N P95** |
| --- | --- | --- | --- | --- | --- | --- | --- | --- |
| Full Model | 4.54 | 0.96 | 1.30 | 6.12 | 112.07 | 12.41 | 16.74 | 132.48 |
| No Environmental Penalty (($\lambda$ = 0)) | 4.71 | 1.18 | 1.59 | 6.65 | 118.62 | 17.95 | 24.21 | 148.15 |
| No Abstention | 4.57 | 1.07 | 1.44 | 6.33 | 113.84 | 15.22 | 20.53 | 138.88 |
| Weather Only | 3.98 | 1.31 | 1.77 | 6.13 | 109.26 | 19.88 | 26.82 | 141.96 |
| Soil and Terrain Only | 4.21 | 1.22 | 1.65 | 6.22 | 111.45 | 18.03 | 24.32 | 141.11 |
| Crop Indices Only | 4.09 | 1.27 | 1.71 | 6.18 | 110.03 | 18.76 | 25.31 | 140.89 |

# Table S18b Panel

| **Coverage Level** | **Mean Profit** | **Profit Std.** | **Profit IQR** | **Abstain (%)** | **N Std.** | **N IQR** | **N P95** |
| --- | --- | --- | --- | --- | --- | --- | --- |
| 0.90 | 4.61 | 1.08 | 1.46 | 18.4 | 15.37 | 20.73 | 136.00 |
| 0.95 | 4.54 | 0.96 | 1.30 | 27.9 | 12.41 | 16.74 | 132.48 |

# Table S19

| **Ref** | **UQ signal** | **Decision rule** | **FS guarantee** | **Abstain** | **Key reported outcome** |
| --- | --- | --- | --- | --- | --- |
| [15] | Point yield model | Point EONR mapping | No | No | EONR comparison via model fit and ex-post evaluation |
| [36] | Model averaging uncertainty | Point EONR estimation | No | No | Improved ex-post EONR estimation without selective action |
| [48] | Quantile or RF-style spread | Prediction intervals only | No | No | Yield-level uncertainty without prescription reliability |
| [43, 49] | Conformal prediction intervals | Prediction intervals only | Yes | No | Calibrated prediction coverage, not calibrated prescriptions |
| [44] | Conformalized prescriptive ML | Decision-aware optimization | Yes | No | Decision-aware conformalization outside the nitrogen-management context |
| [37] | Reject-option theory | Abstain or predict | No | Yes | Formal abstention not linked to agronomic profit |
| This work | Split conformal yield intervals | Profit LCB optimization | Yes | Yes | Abstention of 18.4% (0.90) and 27.9% (0.95), with stable prescriptions ((\sigma_N = 12.41), (\sigma_\pi = 0.96)) |
